# Targeting *Plasmodium falciparum* with purine antimetabolites as a therapeutic strategy

**DOI:** 10.3389/fmicb.2026.1773050

**Published:** 2026-03-17

**Authors:** Worlanyo Tashie, Harry P. de Koning, Nancy O. Duah-Quashie, Neils B. Quashie

**Affiliations:** 1 West African Centre for Cell Biology of Infectious Pathogens, Department of Biochemistry, Cell and Molecular Biology, College of Basic and Applied Sciences, University of Ghana, Accra, Ghana; 2 School of Infection and Immunity, College of Medical, Veterinary and Life Sciences, University of Glasgow, Glasgow, United Kingdom; 3 Department of Epidemiology, Noguchi Memorial Institute for Medical Research, University of Ghana, Accra, Ghana; 4 Centre for Tropical Clinical Pharmacology and Therapeutics, University of Ghana Medical School, Accra, Ghana

**Keywords:** drug discovery, drug transporter, nucleotide metabolism, P*f* ENT, plasmodium falciparum, purine antimetabolites

## Abstract

*Plasmodium falciparum* lacks the *de novo* purine biosynthesis pathway and relies exclusively on salvaging free purines from the host to meet its metabolic requirements. This absolute dependence on the purine salvage pathway provides a compelling opportunity for antimalarial drug development, particularly in the face of rising resistance to current therapies. Although the purine salvage system has been extensively studied as a potential drug target in *P. falciparum*, no purine-based antimalarial drug has yet reached clinical use. In this review, we summarize the potential of targeting the purine salvage pathway in antimalarial drug development, with a focus on strategies that leverage *P. falciparum* Equilibrative Nucleoside Transporters (*Pf* ENTs) as conduits for therapeutic agents. Purine analogs that efficiently enter *P. falciparum*-infected erythrocytes, reach *Pf* ENTs, and undergo selective activation within the parasite can disrupt purine metabolism and nucleic acid synthesis, ultimately leading to parasite death. The *Pf* ENTs therefore offer a unique and viable route for delivering purine-based analogs into the parasite. Such approaches provide a framework for target-based design of purine-analog-based antimalarial therapies.

## 1 Introduction

Malaria continues to pose a major public health challenge, with sub-Saharan Africa bearing the highest burden (Phillips et al., 2017; WHO, 2025c). In 2024, an estimated 282 million cases of malaria were reported worldwide, resulting in 579,000 deaths. Africa is the continent most affected and accounts for approximately 94% of all malaria cases (WHO, 2025c). Among the five *Plasmodium* species that infect humans, *Plasmodium falciparum* is the most virulent and is responsible for the majority of malaria-related deaths (Daily and Parikh, 2025; WHO, 2025c).

Chemotherapy remains a cornerstone of malaria control. However, the increasing resistance of *P. falciparum* to existing antimalarial drugs poses a significant threat to global efforts to control and eradicate the disease (Theodoridis and Carvalho, 2025; WHO, 2025c). In response to the growing problem of resistance, the World Health Organizations (WHO) recommended artemisinin-based combination therapies (ACTs) as the first-line treatment for uncomplicated malaria, which have since become the cornerstone of malaria case management (Nosten and White, 2007; WHO, 2025b). The success of ACTs lies in their pharmacological synergy: a rapidly acting artemisinin derivative which has a short plasma half-life is combined with a longer acting partner drug. This strategy limits the parasite's exposure time to the artemisinin, thereby reducing the likelihood of developing resistance (Kavishe et al., 2017). Nevertheless, the efficacy of ACTs is increasingly threatened by the emergence and spread of artemisinin resistance, first reported in Western Cambodia in the late 2000s (Dondorp et al., 2009; Noedl et al., 2008), but now increasingly reported from a much wider geographical area (Ward et al., 2022). Artemisinin partial resistance is characterized by delayed clearance of *P. falciparum* from the bloodstream following treatment with artemisinin-based therapies. Accumulating evidence demonstrates that mutations in the *PfKelch13* propeller domain (*PfK13*) are associated with this delayed parasite clearance both *in vitro* and *in vivo* after artemisinin treatment (Rosenthal et al., 2024; WHO, 2025a). In Africa, artemisinin partial resistance has been confirmed in multiple countries, including Rwanda (Straimer et al., 2022; Uwimana et al., 2021), Uganda (Balikagala et al., 2021; Conrad et al., 2023; Ogwang et al., 2024), Eritrea (Mihreteab et al., 2025), and Tanzania (Juliano et al., 2024). These artemisinin partial resistant parasites have emerged independently and have not spread from South-East Asia (WHO, 2025a). Although ACTs remain highly effective in most African settings (Assefa et al., 2024), the recent emergence of partial resistance to artemisinin-based therapies, including combinations, in parts of Africa underscores the urgent need for novel therapeutic antimalarial strategies.

One promising strategy lies in targeting the parasite's absolute dependency on host-derived purines for survival. Purines and pyrimidines are essential for the synthesis of DNA, RNA, and are critical metabolites in all living organisms (el Kouni, 2017; Krungkrai and Krungkrai, 2016). Unlike humans, who can synthesize purine nucleotides both *de novo* and through salvage pathways, *Plasmodium* species lack the *de novo* purine biosynthesis pathway (de Koning et al., 2005; Webster et al., 1984) but do synthesize pyrimidines *de novo* (Cassera et al., 2011a; de Koning et al., 2005). Consequently, *P. falciparum* relies entirely on salvaging purines precursors from its host to meet metabolic demands (Campagnaro and de Koning, 2020; Gero and O'Sullivan, 1990; Webster et al., 1984). This unique dependence represents a critical vulnerability in the parasite's biology and offers a valuable target for antimalarial drug development.

The uptake of purines into the parasite's cytosol, mediated by Equilibrative Nucleoside Transporters (*Pf* ENTs), is a critical step in the purine salvage pathway (Campagnaro and de Koning, 2020; Carter et al., 2003; Riegelhaupt et al., 2010). Compromising this pathway could effectively deprive the parasite of its essential purine supply and subsequently lead to its death. A promising strategy is to exploit the *Pf* ENTs as conduits for delivering drugs into the parasite. This approach would allow for the inhibition of essential enzymes or the disruption of nucleic acid synthesis, leading to chain termination or lethal mutagenesis. *Pf* ENT1 is a high affinity, high capacity transporter and most likely energy-dependent, and therefore directs effective, monodirectional and concentrative uptake of purines and their analogs into the parasite. Its broad specificity for nucleosides and bases doubtlessly aids in the design of antimalarial purine antimetabolites.

While the purine salvage system has been extensively studied as potential drug targets in *P. falciparum* (Cheviet et al., 2019; Frame et al., 2015a; Quashie et al., 2008), no purine-based antimalarial drug has yet reached clinical application or even, to the best of our knowledge, clinical development. This is in large part because efforts have centered more on the development of inhibitors of key enzymes of *P. falciparum* purine metabolism, including transition-state inhibitors of *P. falciparum* purine nucleoside phosphorylase (*Pf* PNP) such as DADMe-Immucillin-G (Cassera et al., 2011b) and 5′-methylthio-immucillin-H (Ting et al., 2005), as well as inhibitors targeting *P. falciparum* hypoxanthine-guanine-xanthine-phosphoribosyl transferase (*Pf* HGXPRT), rather than on the challenges of delivering these analogs into the infected human erythrocyte and thence into the parasite.

This review focuses on the untapped potential of *Pf* ENTs as therapeutic targets for inhibitors and as conduits for purine antimetabolites, emphasizing their critical role in the parasite's survival and the opportunities they offer for the development of purine-based antimalarial strategies. With the escalating threat of drug-resistant malaria, and the implementation of a truly effective vaccine always fading into the future (Sibomana et al., 2025), innovative approaches such as these are urgently needed to strengthen global efforts to combat this deadly disease.

## 2 Purine transport and metabolism in *P. falciparum*

### 2.1 Transport of purines across biological membranes

Purine nucleobases and nucleosides are hydrophilic and hence cannot freely cross the lipid bilayer of plasma membranes (Campagnaro and de Koning, 2020; Carter et al., 2003). This makes the internalization of purine nucleobases and nucleosides dependent on specialized transport systems to facilitate their import into, or export out of, prokaryotic and eukaryotic cells. The type of purine transporter present in cellular membranes determines the purine nucleobases and nucleosides that are transported across it (Campagnaro and de Koning, 2020; Rehan et al., 2019).

In protozoa, these can be specific for one or more nucleosides (Al-Salabi et al., 2007; Carter et al., 2000b), for nucleobases (Aldfer et al., 2024; Burchmore et al., 2003), or both (de Koning and Jarvis, 1999). Moreover, protozoan transporters can be purine-specific, pyrimidine specific, or mixed purine/pyrimidine transporters (Aldfer et al., 2022a; Alzahrani et al., 2017; de Koning and Jarvis, 1999; Elati et al., 2023). Many studies have implicated protozoan ENT transporters in the uptake of purine antimetabolites (Aldfer et al., 2022a; Al-Salabi and de Koning, 2005; Hulpia et al., 2019a; Ungogo et al., 2023; Vasudevan et al., 1998) and/or other drugs, including the diamidine and melaminophenyl arsenical trypanocides (Carter et al., 1999; de Koning et al., 2000a; Stewart et al., 2010). This has also been demonstrated for *P. falciparum* (Cassera et al., 2011b; Sosa et al., 2019; Tashie et al., 2025b; Ting et al., 2005) and will be discussed in section 3.2, below.

The expression levels of protozoan ENT transporters are regulated by various factors such as substrate availability, cell cycle phase and life cycle stage (de Koning et al., 2000b; Hall et al., 1996; Martin et al., 2014; Menezes et al., 2025). Where this has been studied, protozoan nucleoside and nucleobase transporters have been found to be proton symporters (de Koning and Jarvis, 1997, 1998; de Koning et al., 1998; Landfear et al., 2004; Ortiz et al., 2009), which enables them to efficiently transport substrate at low concentrations and against a concentration gradient. These are ideal characteristics for an antimetabolite transporter.

### 2.2 Purine salvage in *P. falciparum*

The genome of *P. falciparum* is rich in adenine (80.6% A-T content), and replication thus requires a very efficient supply of adenine nucleotides (Cheviet et al., 2019; Gardner et al., 2002), especially given the very high rate of replication in the human host. In the intraerythrocytic stages of *P. falciparum*, only one of the ten enzymes required for *de novo* synthesis of purine, namely adenylosuccinate lyase, has been identified (Krungkrai and Krungkrai, 2016) and this is used in the conversion of inosine monophosphate to AMP. Consequently, the parasite, being unable to synthesize purines *de novo*, must import purines into its cytosol at a very high rate to meet its purine requirements. The low concentration of free purine nucleosides and nucleobases in the erythrocyte cytosol necessitates high substrate affinity and the required quantity necessitates a high rate of uptake (*V*_*max*_); nucleotides such as ADP, ATP, and GTP are not taken up across the plasma membrane. The combination of high affinity and high capacity in turn necessitates a form of active transport, likely in the form of proton symport as shown for other protozoan purine transporters (see above—section 2.1).

The import of purine nucleobases (adenine, guanine, hypoxanthine, and xanthine) and nucleosides (adenosine, guanosine, and inosine) into the parasite's cytosol is mediated by the *Pf* ENTs. Genome analysis reveals four *Pf* ENTs: *Pf* ENT1, *Pf* ENT2, *Pf* ENT3, and *Pf* ENT4 (Fox and Bzik, 2020; Frame et al., 2015a; Ghérardi and Sarciron, 2007). *Pf* ENT1 and *Pf* ENT4 are localized in the parasite's plasma membrane (Frame et al., 2012, 2015a), while *Pf* ENT2 localizes to the endoplasmic reticulum (Frame et al., 2015a); the localization of *Pf* ENT3 has not yet been reported. All *Pf* ENTs are expressed during the intraerythrocytic stages of the parasite's life cycle (Frame et al., 2012, 2015a; Ghérardi and Sarciron, 2007; Quashie et al., 2008).

**Figure 1:**
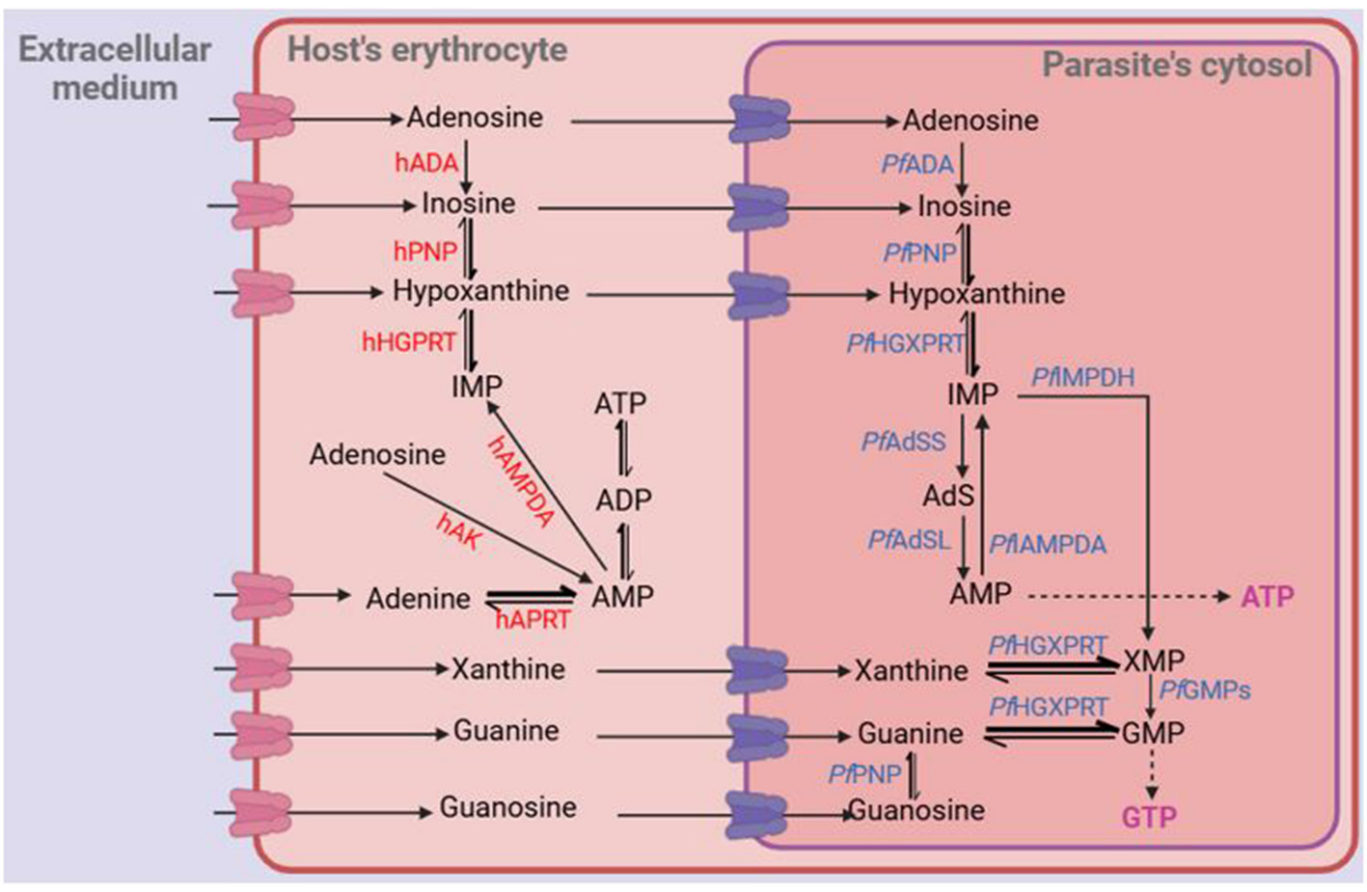
Purine salvage in *P. falciparum*. Bold arrows on reversible steps indicate the metabolically favored direction. hADA, human adenosine deaminase; hPNP, human purine nucleoside phosphorylase; hHGPRT, human hypoxanthine-guanine phosphoribosyl transferase; hAK, human adenosine kinase; hAMPDA, human adenosine 5′-monophosphate deaminase; hAPRT, human adenine phosphoribosyl transferase; AMP, adenosine 5′-monophosphate; ADP, adenosine 5′-diphosphate; ATP, adenosine 5′-triphosphate; IMP, inosine 5′-monophosphate; XMP, xanthosine 5′-monophosphate; GMP, guanosine 5′-monophosphate; AdS, adenylosuccinate; PfADA, *P. falciparum* adenosine deaminase; PfPNP, P. falciparum purine nucleoside phosphorylase; *Pf* HGXPRT, *P. falciparum* hypoxanthine-guanine-xanthine phosphoribosyl transferase; *Pf* AMPDA, *P. falciparum* adenosine 5′-monophosphate deaminase; *Pf* IMPDH, *P. falciparum* inosine 5′-monophosphate dehydrogenase; *Pf* GMPs, *P. falciparum* guanosine 5′-monophosphate synthase; *Pf* AdSS, adenylosuccinate synthetase; *Pf* AdSL, adenylosuccinate lyase. Adapted from Ducati et al. (2013).

Following the import of purine nucleobases and nucleosides into the parasite's cytosol, their incorporation into the nucleotide pool is mediated by a set of well-characterized purine metabolism enzymes. *Pf* HGXPRT converts salvaged hypoxanthine, guanine and xanthine into inosine monophosphate (IMP), guanosine monophosphate (GMP) and xanthosine monophosphate (XMP), respectively. *Pf* PNP convert nucleosides into their corresponding nucleobases and sugar counterparts, thereby funneling inosine, xanthosine, and guanosine as nucleobases through *Pf* HGXPRT, yielding the corresponding mononucleotides. Unlike many other protozoa (Vodnala et al., 2008) including the apicomplexan *Toxoplasma gondii* (Kim et al., 2008), *P. falciparum* cannot directly convert adenosine to AMP, because it lacks the gene for adenosine kinase, and must undergo a two-step conversion to hypoxanthine instead: *P. falciparum* adenosine deaminase (*Pf* ADA) catalyzes the deamination of adenosine into inosine, followed by the phosphorolysis of inosine to hypoxanthine by *Pf* PNP (Cassera et al., 2011a; Cheviet et al., 2019; Ducati et al., 2013; Krungkrai and Krungkrai, 2016). The overall purine salvage pathway in *P. falciparum* is depicted in Figure 1, and has been extensively reviewed by Cassera et al. (2011a), Ducati et al. (2013), and Ginsburg (2016).

The development of purine-based antimalarial drugs must consider the unique purine metabolism in the *Plasmodium* parasite. As shown in Figure 1, *P. falciparum* only incorporates oxopurine nucleobases directly into its nucleotide pool, through a streamlined one-step phosphorylation catalyzed by *Pf* HGXPRT. In contrast, nucleosides, if they are to become nucleotides, must first be catabolised into their respective nucleobases by *Pf* PNPs, introducing an additional and potentially rate-limiting step (Cassera et al., 2011a; Cheviet et al., 2019; Ducati et al., 2013) during which any modifications to the ribose moiety are lost. In addition, adenosine analogs would first have to be deaminated to inosine analogs while antiprotozoal analogs with modifications of the purine ring [e.g., 7-deaza (Hulpia et al., 2019b) or 2-F (Vodnala et al., 2013)] may not be ADA substrates. However, if the adenosine analogs are ADA substrates, they are broken down in human serum (Rottenberg et al., 2005). Therefore, to effectively target the purine salvage pathway in antimalarial drug design, nucleobases should be prioritized over nucleosides due to their direct and efficient incorporation into the parasite's nucleotide pool. This approach was demonstrated by Tashie et al. (2025b) where the authors demonstrated that nucleobases, particularly guanine derivatives, inhibited the *in vitro* growth of *P. falciparum* parasites.

### 2.3 *Pf*ENTs as drug targets in *P. falciparum*

*Pf* ENTs represent a critical link in the parasite's purine salvage pathway and can therefore be exploited in the development of therapeutic agents. Among the *Pf* ENTs, *Pf* ENT1 serves as the primary route for purine uptake in *P. falciparum* and is essential for the parasite's intraerythrocytic survival, validating it as a promising target for antimalarial drug development El Bissati et al., (2006), (2008); Zhang et al., (2018).

Using targeted gene manipulation approaches, El Bissati et al. (2006) and El Bissati et al. (2008) provided compelling genetic evidence for the essential role of *Pf* ENT1 in the parasite's purine salvage pathway. Transgenic parasites lacking *Pf* ENT1 (Δ*Pf* ENT1) exhibited a conditionally lethal growth phenotype under physiological purine concentrations, demonstrating an inability to complete the intraerythrocytic life cycle when hypoxanthine, inosine, adenosine, xanthine, guanine or guanosine were provided at concentrations sufficient to support wild-type parasite growth (El Bissati et al., 2006, 2008). Reintroduction of *Pf* ENT1 into the knock-out background fully restored purine utilization and growth across a broad range of nucleobases and nucleosides, confirming that the observed phenotype was attributable specifically to loss of *Pf* ENT1 rather than secondary genetic effects (El Bissati et al., 2008). Together, these findings establish *Pf* ENT1 as an essential and primary purine uptake route in *P. falciparum*, providing strong experimental validation of *Pf* ENT1 as a potential drug target in the parasite.

In addition to genetic validation, pharmacological evidence further supports *Pf* ENT1 as a viable drug target in the parasite. Studies by Frame et al. (2015b) and Sosa et al. (2019) identified small-molecule inhibitors of *Pf* ENT1 that inhibited the *in vitro* growth of *P. falciparum*, providing direct functional evidence for the therapeutic potential of targeting purine transport in *P. falciparum*.

Despite being the primary purine uptake route in the parasite, with a high rate of substrate translocation, various studies, employing different expression systems and experimental methodologies to characterize *Pf* ENT1, as well as slightly different primary sequence, have yielded inconsistent purine uptake kinetics, fueling ongoing scientific debate about its substrate preference (Table 1). Among the main contributors of the variability is the expression in *Xenopus laevis* oocytes compared to studies using live *P. falciparum* trophozoites freshly isolated from human erythrocytes (without codon optimisation); we have ourselves attempted the expression of *Pf* ENTs including *Pf* ENT1 in other protozoa, after optimizing the highly AT-rich sequences for the codon preferences of the heterologous expression cells, and found the results insufficiently reliable (unpublished data) although we have been highly successful with ENT and non-ENT transporters from other organisms [e.g., Aldfer et al., 2022b; Natto et al., 2021b]. More important is the difference in assay conditions. Whereas the studies by Downie et al. (2006), Downie et al. (2008), Parker et al. (2000), and Carter et al. (2000a) used high radiopermeant concentrations (10–100 μM), our earlier work (Quashie et al., 2008) utilized far lower concentrations of radiolabelled permeant (30–250 nM), allowing the detection rather than saturation of high affinity transporters. Moreover, the saturating radiolabel concentrations cause the transporter to operate at *V*_*max*_ and may thereby saturate the downstream metabolic enzymes as well, causing the permeant to back up and exceed the extracellular concentration. In contrast, we utilized the lowest possible [^3^H]-permeant concentrations, which allowed the accurate determination of a sub-micromolar *K*_*m*_ value for hypoxanthine and low micromolar *K*_*m*_ for adenosine—reasonable values given the low concentration of free purines in freshly obtained human erythrocytes [~10 nM (Casali et al., 2016)] and the sub-micromolar *K*_*m*_ value of *Pf* HGXPRT for hypoxanthine [0.46 μM (Queen et al., 1988); 0.9 μM (Keough et al., 1999)]. Proposed substrate *K*_*m*_ values in the 100s of μM for *Pf* ENT1 make no physiological sense as a transporter with a *K*_*m*_ of 250 μM would operate at just 0.04% of *V*_*max*_ at a substrate concentration of 0.1 μM, or 0.4% at a concentration of 1 μM. Moreover, all other protozoan nucleoside and nucleobase transporters yet identified are of the ENT family and display sub-micromolar to low micromolar *K*_*m*_ values for their primary substrates (Aldfer et al., 2022a),b; Campagnaro and de Koning, 2020; de Koning et al., 2005; Elati et al., 2023; Landfear et al., 2004).

**Table 1:** Reported purine uptake kinetics of *Pf* ENT1 across different experimental systems.

**Substrate or inhibitor**	***Xenopus laevis*** **oocytes**				**Isolated trophozoites-stage of** ***P. falciparum***		
	**Carter et al. (2000a)**	**Parker et al. ((2000))**	**Downie et al. (2006)**	**Downie et al. (2008)**	**Downie et al. (2006)**	**Quashie et al. (2008)**	**Downie et al. (2008)**
***K**_*m*_* **value (**μ**M)**							
Hypoxanthine		410				0.34	
Adenosine	13.2	320	1,860		1,450	2.0	
Inosine	253						
Adenine		320		820			1,200
Thymidine			1,330		1,110		
Uridine		3,500					
***K**_*i*_* **value (**μ**M)**							
Hypoxanthine		##				0.75^1^	
Guanine	–	###				0.11^2^	
Adenosine		##				4.0^2^	
Inosine		#				2.0^1^	
Adenine	–	##				240^1^	
Guanosine	++	#				11.6^2^	
Thymidine	+	#					
Uridine	+/–	#					

“–” denotes no reported K_m_ data. ^1^[^3^H]-adenosine permeant. ^2^[^3^H]-hypoxanthine permeant. –, +/–, +, ++: level of inhibition form none (–) to strong (++) in an assay with 500 μM inhibitor and 5 μM [^3^H]-adenosine. #, ##, ###: cumulative uptake of 100 μM permeant in X. laevis expressing PfENT1, from modest (#) to strong (###).

Nevertheless, converging evidence suggests that *P. falciparum* relies primarily on high-affinity oxopurine nucleobase transport, with hypoxanthine as its major purine source. This inference is supported by the observation that depletion of hypoxanthine from the culture medium using the enzyme xanthine oxidase prevents parasite growth (Berman et al., 1991); the standard supplementation of *P. falciparum* culture media with hypoxanthine (Basco, 2023; Tewari et al., 2019); and early biochemical studies demonstrating efficient incorporation of hypoxanthine into the parasite's nucleic acids (Büngener and Nielsen, 1968; Gutteridge and Trigg, 1970). Collectively, these findings reinforce the parasite's dependence on nucleobase transport for survival and underscore the relevance of *Pf* ENTs, and especially *Pf* ENT1, as a drug target. Findings from these studies further suggest that *Pf* ENT1 is a broad-specificity purine transporter, and its broad substrate selectivity/permissibility supports the delivery of purine antimetabolites through this transporter.

### 2.4 Purine transport in uninfected and *P. falciparum*-infected human erythrocytes

The mature human erythrocyte, which provides the host environment for *P. falciparum* growth and multiplication during the intraerythrocytic stages, lacks the enzymatic machinery necessary to synthesize purine rings *de novo*. Consequently, erythrocytes rely on salvaging preformed purine nucleobases and nucleosides from the plasma or by degrading purine nucleotides and nucleosides within the erythrocytes to meet their purine requirements (Dudzinska et al., 2006).

The human plasma contains purines at micromolar concentrations, with hypoxanthine (1–5 μM) and inosine (~1 μM) being the predominant forms. Adenosine is also found in the human plasma and ranges from nanomolar values up to 1–5 μM. The total concentration of purines in the plasma is below 10 μM (Campagnaro and de Koning, 2020; Frame et al., 2015a). Hypoxanthine, inosine, adenosine and other purines found in the erythrocytes originate from uptake by purine transporters and by the metabolism of erythrocyte ATP, which is present at approximately 2 mM (Frame et al., 2015a).

The steady-state purine concentration in human erythrocytes is not sufficient to support the multiple rounds of *P. falciparum* DNA replication that occur during the 48-h intraerythrocytic stage of the parasite's life cycle, giving rise to 16–32 merozoites (Josling and Llinás, 2015). It has been estimated that the erythrocyte's complement of ATP would be sufficient for only 1%−2% of the parasite's purine requirements for nucleic acid synthesis alone. Thus, large quantities of purine nucleobases and nucleosides must be imported into the *P. falciparum*-infected erythrocytes to supply enough purines to the developing intracellular parasite. The import of purines into the erythrocyte's cytosol is mediated through two main pathways: nucleosides enter through human ENT1 (hENT1) while nucleobases primarily enter via human facilitated nucleobase transporter (hFNT1; Baldwin et al., 2007; Berman et al., 1991; Domin et al., 1988; Frame et al., 2015a; Wallace et al., 2002).

Because the *P. falciparum* parasite is far more metabolically active than its host erythrocyte, the rate at which some key nutrients are consumed exceeds the rate at which they can be taken up by erythrocyte transporters (Counihan et al., 2021). To accommodate this increased metabolic demand, *P. falciparum* alters the permeability of the host erythrocyte membrane either by up-regulating existing host transport systems and/or by inducing parasite-encoded “new permeation pathways” that are inserted into the host cell plasma membrane (Counihan et al., 2021; Ginsburg et al., 1985; Thomas and Egée, 2005). These new permeation pathways are induced approximately 10–20 h after parasite invasion and allow for entry of broad range of nutrients necessary for parasite growth and survival (Ginsburg et al., 1985; Lingelbach et al., 2004). However, despite their substantial contribution to nutrient uptake in infected erythrocytes, a study by Quashie et al. (2010) demonstrated that the host erythrocyte transporters hENT1 and hFNT1, rather than the new permeation pathways, serve as the primary route for entry of adenosine, adenine and hypoxanthine into *P. falciparum*-infected human erythrocytes. Both human transporters were upregulated in the infected erythrocytes, leading to 2–2.5-fold higher rates of uptake of [^3^H]-adenosine and [^3^H]-hypoxanthine (Quashie et al., 2010).

## 3 Therapeutic potential of purine analogs

### 3.1 Nucleobase and nucleoside analogs as chemotherapeutic agents

Nucleobase and nucleoside analogs are mostly synthetic, chemically modified compounds designed to mimic the physiological properties of naturally occurring nucleobases and nucleosides. By mimicking these molecules, nucleobase and nucleoside analogs exploit existing cellular pathways for transport, activation, and conversion into active phosphate derivatives, which disrupt cellular processes (Hruba et al., 2023; Jordheim et al., 2013; Rehan et al., 2019).

Analogs of the naturally occurring purine nucleobases and nucleosides, modified at the nucleobase, ribose or phosphate moieties to enhance therapeutic potency and selectivity are among the most potent anticancer, antibacterial and antiviral agents. Modifications to the nucleobase often involve halogenation, azotation, N-conjugation of the base, or the introducing a different heterocyclic ring or acyclic moiety for (2′-deoxy)ribose; changes such as replacing oxygen with another atom, altering ring configurations, or substituting with acyclic fragments expand therapeutic applications. The phosphate group may be protected, substituted with another phosphorus-containing group (e.g., phosphonate) or a fragment from another compound (such as an amino acid), or entirely eliminated. These modifications have enabled the development of a wide range of analogs with therapeutic applications in clinical settings (Jordheim et al., 2013; Pastuch-Gawołek et al., 2019; Thomson and Lamont, 2019).

Due to the charged nature of nucleotides, they cannot cross the lipid bilayer of the plasma membrane. Hence, purine or pyrimidine analogs designed as therapeutic agents are administered as prodrugs, requiring sequential phosphorylation catalyzed by target cell nucleo(s/t)ide kinases to transform them into their mono-, di-, and tri-phosphorylated active forms. Once activated, the activated antimetabolites exert therapeutic effects through several mechanisms, including inhibition of key intracellular enzymes like viral DNA or RNA polymerases; interference with wider nucleotide metabolism including activated intermediates; incorporation into nucleic acid chains which eventually leads to termination of the DNA or RNA replication or altered structure; promotion of lethal mutagenesis by inducing errors in viral genomes to render them non-viable; and depletion of natural nucleotide pools to starve the cell or pathogen of essential precursors (Ali et al., 2013; Borbone et al., 2021; Galmarini et al., 2002; Kamzeeva et al., 2023; Pruijssers and Denison, 2019; Rehan et al., 2019; Thomson and Lamont, 2019).

### 3.2 Applications of nucleobases and nucleosides analogs as antimalarial agents

While primarily known for their use in treating cancers (Huang et al., 2022; Muggia et al., 2012) and viral infections (Feng, 2018; Geraghty et al., 2021; Pastuch-Gawołek et al., 2019), nucleobase and nucleoside analogs are also effective against parasitic diseases. Studies have highlighted their activity against *Trypanosoma* (Fiuza et al., 2022; Hulpia et al., 2019b; Mabille et al., 2022), *Leishmania* (Lin et al., 2022), *Toxoplasma* (Al Safarjalani et al., 2008; Elati et al., 2023), *Trichomonas* (Natto et al., 2021a) and *Plasmodium* (Sosa et al., 2019; Tashie et al., 2025b; Ting et al., 2005) parasites. These analogs interfere with parasite-specific enzymes or nucleoside transporters, thus offering a targeted approach to antiparasitic therapy (Elati et al., 2023; Fiuza et al., 2022; Hulpia et al., 2019b; Natto et al., 2021a; Sosa et al., 2019; Ting et al., 2005).

Several purine nucleobase and nucleoside analogs have shown potent activity against *P. falciparum* parasites. Sosa et al. (2019) identified six inhibitors of *Pf* ENT1 as potential therapeutic leads for antimalarial drug development. Similarly, Cassera et al. (2011b) demonstrated that the transition state nucleoside analog DADMe-Immucillin-G (BCX4945) effectively killed *P. falciparum* by inhibiting *Pf* PNP. Another transition state analog, 5′-methylthio-immucillin-H, exhibited selective inhibition of *Pf* PNP over the human enzyme, resulting in parasite death *in vitro* (Ting et al., 2005). More recently, Tashie et al. (2025b) demonstrated that the guanine derivatives 8-azaguanine, 7-deazaguanine, and 6-thioguanine significantly inhibited the *in vitro* growth of *P. falciparum*. Their findings highlight these nucleobase analogs as promising leads for purine-based antimalarial drug development and underscore the versatility of *Pf* ENTs in the uptake of purine antimetabolites (Tashie et al., 2025b).

## 4 Targeting *Pf*ENTs with purine analogs

Purine analogs targeting the *Pf* ENTs can act as inhibitors of purine metabolism enzymes or as “subversive substrates” that become toxic following activation by the parasite's purine salvage enzymes (Berg et al., 2010; el Kouni, 2003; Hulpia et al., 2019a). Although purine analogs could be designed to inhibit *Pf* ENTs directly, this approach may present with some challenges. Transporter inhibitors typically have a transient association with the target and require sustained concentrations to compete effectively, and for an extended time, with endogenous substrates—hypoxanthine and adenosine in the case of *Pf* ENT1. It is unlikely that depriving the parasites of purines for a short duration would do irreversible damage, and certainly this is not the case in kinetoplastids (de Koning et al., 2000b; Martin et al., 2014), although a prolonged absence of purine salvage is certainly lethal.

Ideally, novel antimalarial agents directed at *Pf* ENT1 should exhibit broad-spectrum activity against all human malaria parasites. Chen et al. (2025) and Deniskin et al. (2016) characterized the substrate specificity and transport kinetics of *Pf* ENT1 orthologs in *P. vivax* (*Pv*ENT1) and the rodent malaria *P. berghei* (*Pb*ENT1), respectively, reporting distinct substrate recognition profiles. Arora et al. (2016) expressed *Pb*ENT1 in yeast and characterized it as a nucleoside/base transporter with broad specificity and highest affinity for inosine. Two separate studies have reported characterisations of *Pv*ENT1, both reporting highest affinity for inosine followed by guanosine, albeit with an order of magnitude difference in substrate affinities between the two reports (Chen et al., 2025; Deniskin et al., 2016). Although differences in substrate affinities have been reported among *Pf* ENT1 orthologs, purine inhibitors shown to act on *Pf* ENT1 were also effective against *Pv*ENT1 (Chen et al., 2025; Deniskin et al., 2016) and *Pb*ENT1 (Arora et al., 2016). This supports the feasibility of developing *Pf* ENT1-targeted purine-based antimalarials with activity across multiple *Plasmodium* species. Nevertheless, a more comprehensive characterization of *Pf* ENT1 orthologs in human non-falciparum *Plasmodium* species would be beneficial in informing the target-based design and optimisation of such agents.

**Figure 2:**
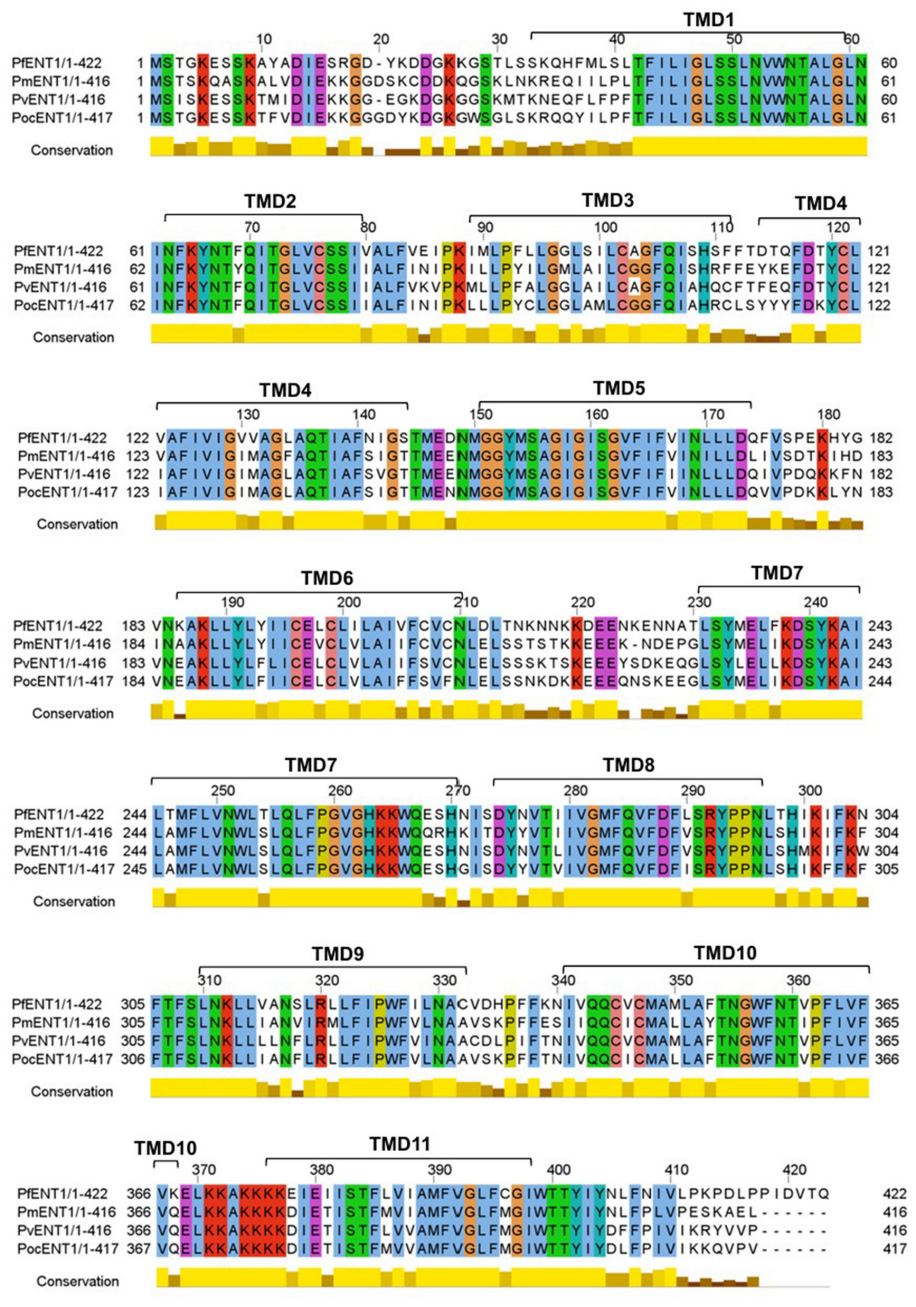
Multiple sequence alignment of ENT1 orthologs annotated with the 11 transmembrane domains (TMD1−11). Sequences included are from *P. falciparum* (*Pf* ENT1; PlasmoDB ID: PF3D7_1347200), *P. malariae* (*Pm*ENT1; PlasmoDB ID: PmUG01_12017700), *P. vivax* (*Pv*ENT1; PlasmoDB ID: PVP01_1207600) and *P. ovale curtisi* (*Poc*ENT1; PlasmoDB ID: PocGH01_12015900). Predicted transmembrane domains are indicated above the alignment and were defined based on *Pf* ENT1 and mapped across orthologs. The conservation histogram below each alignment block represents the degree of residue conservation across species, with higher bars indicating greater conservation. *Pf* ENT1 shares 74.34, 75.0, and 75.42% amino acid sequence identity with *Pm*ENT1, *Pv*ENT1, and *Poc*ENT1, respectively, demonstrating high conservation across human-infecting *Plasmodium* species.

Although no functional data has yet been reported for *Pf* ENT1 homologs in *P. malariae* and *P. ovale*, sequence alignment analyses indicate substantial amino acid identity between *Pf* ENT1 and its orthologs: 74.34% for *P. malariae* and 75.42% for *P. ovale curtisi* (Figure 2). While overall amino acid conservation does not guarantee identical substrate specificity, the high level of similarity, especially across multiple transmembrane domains (TMDs), supports the possibility that purine-based antimetabolites targeting *Pf* ENT1 may have cross-species activity. Consistent with this, *Pv*ENT1 also shares 75% amino acid identity with *Pf* ENT1, and *Pf* ENT1-targeting antimetabolites have demonstrated inhibitory activity against *Pv*ENT1 (Chen et al., 2025; Deniskin et al., 2016). As can be seen in Figure 2, there are no major differences in amino acid sequence within the TMDs of the four *Plasmodium* ENT1s, such as the introduction of a polar residue in the middle of a TMD, which would be indicative of a potential change in the substrate-binding site. Indeed, it has been shown that the four residues most involved in interaction with *Pf* ENT1 substrate inosine are Trp53 (TMD1), Gln135 (TMD4), Asp287 and Arg291 (both TMD8; Wang et al., 2023), which are all unchanged in the various *Plasmodium* ENT1s (Figure 2). Together, these findings support the premise that antimetabolites targeting *Pf* ENT1 can achieve broad antimalarial activity across diverse *Plasmodium* species.

**Figure 3:**
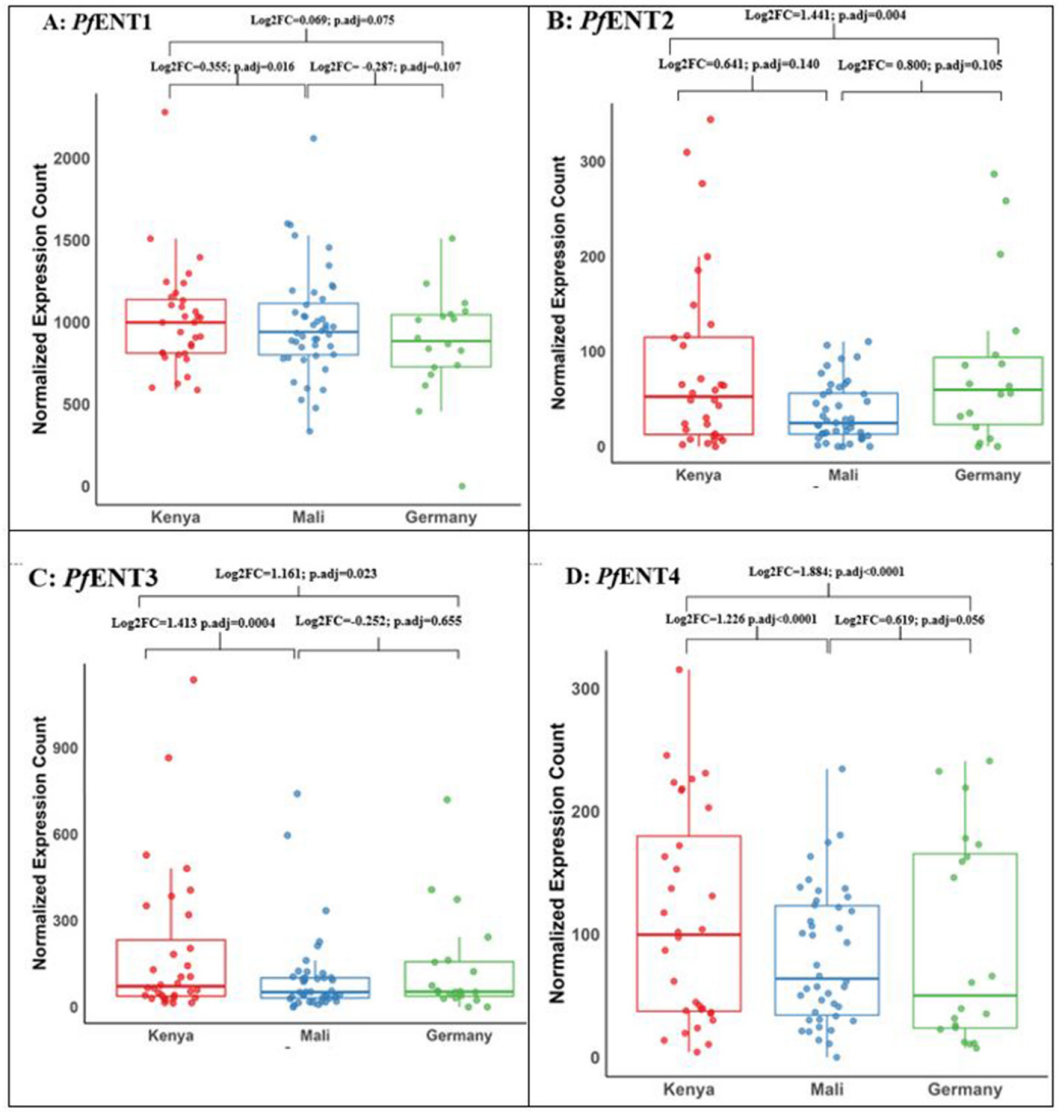
Gene expression profile across distinct geographical locations. **(A)** Graphical representation of gene expression profile in *Pf* ENT1 across distinct geographical locations. **(B)** Graphical representation of gene expression profile in *Pf* ENT2 across distinct geographical locations. **(C)** Graphical representation of gene expression profile in *Pf* ENT3 across distinct geographical locations. **(D)** Graphical representation of gene expression profile in *Pf* ENT4 across distinct geographical locations. The plots above depict the normalized gene expression profiles of all four *Pf* ENTs across three distinct geographical locations: Kenya, Mali, and Germany. Publicly available RNA-seq datasets were used, comprising samples from individuals with uncomplicated *P. falciparum* mono-infections. Kenya dataset: Obtained from Kenyan children, as described by Kimenyi et al. (2024) and available under GEO accession number GSE240643; Mali dataset: Obtained from Malian children, as described by Tebben et al. (2024) and available under BioProject PRJNA962942; Germany dataset: Collected from adult travelers returning to Germany, as described by Wichers et al. (2021) and available under BioProject PRJNA679547. A total of 94 RNA-seq datasets were analyzed: 32 from Kenya, 43 from Mali, and 19 from Germany. The box plots presented here display the normalized expression count of the genes which have been adjusted to account for differences in sequencing depth and other technical variations. This normalization ensures that the expression levels are comparable across all samples. The box plot represents the interquartile range (IQR), with the middle line indicating the median. Log2FC = Log_2_ fold change; p. adj = adjusted *p* value.

The feasibility of exploiting the *Pf* ENTs as conduits for delivering drugs is supported by evidence that all four *Pf* ENTs are expressed in both laboratory strains (Frame et al., 2015a) and field isolates of *P. falciparum* (Tashie et al., 2025a; Figure 3), albeit not to the same extent. The highest expression by far was observed for *Pf* ENT1 in all regions from which isolates were investigated. Moreover, of the four *Pf* ENTs, only *Pf* ENT1 has been shown to be essential (El Bissati et al., 2006, 2008). Essentially is not required for effective drug targeting through a specific transporter, however. For instance, the TbAT1/P2 aminopurine transporter of *T. brucei* mediates the uptake of highly potent purine antimetabolites (Geiser et al., 2005; Hulpia et al., 2019b), as well as several trypanocidal drugs (Lüscher et al., 2007) but is non-essential (Matovu et al., 2003). However, a major advantage of targeting uptake through an essential transporter over a non-essential one is that transporter-related resistance is likely to develop much faster in the latter scenario (Delespaux and de Koning, 2013). In addition, the targeted transporter must be located in the plasma membrane and for *P. falciparum*, this is only known to be the case for *Pf* ENT1 (Frame et al., 2015a) and *Pf* ENT4 (Frame et al., 2012). Interestingly, the study by Tashie et al. (2025a) revealed that both *Pf* ENT1 and *Pf* ENT4 are highly conserved with low genetic diversity, emphasizing their importance and suitability as drug targets. Conversely, mutant alleles of TbAT1/P2 have been identified in both lab strains (Mäser et al., 1999; Stewart et al., 2010) and field isolates (Graf et al., 2013), and are associated with drug resistance (Munday et al., 2015).

Thus, the *Pf* ENTs, particularly *Pf* ENT1, offer a unique and viable pathway for developing purine-based antimalarial drugs. Rather than inhibiting these transporters, the most promising strategy is to focus on leveraging their activity to deliver effective therapeutic agents into the parasite to cause disruption in its purine metabolism and nucleic acid synthesis.

In addition to being a substrate for the *Pf* ENTs, any purine analog targeting the *P. falciparum* purine salvage pathway needs to ensure that the active compound does reach the parasite, located inside human cells—in the case of *Plasmodium*, erythrocytes. In turn, this means that the antimalarial purine analog must be a substrate of nucleoside transporter hENT1, nucleobase transporter hFNT1 and/or the *Plasmodium*-encoded new permeation pathways. The substrate binding structure-activity relationship of hENT1 and hFNT1 have already been described in considerable detail (Vickers et al., 2004; Wallace et al., 2002) and the contribution of human ENT and CNT transporters to the uptake and sensitivity of many anticancer and antiviral nucleosides has been mapped (King et al., 2006; Pastor-Anglada and Pérez-Torras, 2015; Vaskó et al., 2019).

hFNT displays highest affinity for adenine, with a *K*_*m*_ of ~15 μM, but also good affinity for guanine with reported *K*_*m*_ value of 37 μM and *K*_*i*_ of 28 μM (Table 2; Domin et al., 1988; Wallace et al., 2002)–10-fold higher than for hypoxanthine or xanthine (28 ± 2.0 μM vs. 290 ± 21 μM or >150 μM; Wallace et al., 2002). Importantly, this transporter has remarkable affinity for some therapeutic guanine analogs (Table 2), e.g., a *K*_*i*_ value of 7.4 μM for 9-methylguanine and 8.0 μM for 9-deazaguanine (Wallace et al., 2002). This further reinforces the notion that guanine-based antimetabolites constitute the most promising purine leads against *P. falciparum*, as observed by Tashie et al. (2025b). As an example, 7-deazaguanine was the second-most potent nucleobase analog against intraerythrocytic *P. falciparum* in the latter study (EC_50_ = 14.9 μM) and one of the highest affinity substrates of hFNT1 (K_*i*_ = 15 ± 4.8 μM; Table 2).

**Table 2:** Kinetic constants of the main substrates and selected nucleobase analogs of the hFNT nucleobase transporter of human erythrocytes.

**Substrate or inhibitor**	**Domin et al. ((1988))**		**Wallace et al. ((2002))**	
	***K**_*m*_* **(**μ**M)**	***K**_*i*_* **(**μ**M)**	***K**_*m*_* **(**μ**M)**	***K**_*i*_* **(**μ**M)**
Adenine	13 ± 1	12 ± 1	16 ± 4.5	
Guanine	37 ± 2	50 ± 3		28 ± 2
Hypoxanthine	180 ± 12	200 ± 170		290 ± 21
Xanthine				>150
6-thioguanine				95 ± 8
7-deazaguanine				15 ± 5
9-methylguanine				7.4 ± 2
9-deazaguanine				8.0 ± 2
Allopurinol				150 ± 9
2-thioaminopurinol				20 ± 6

It thus follows that an effective purine antimetabolite strategy against *Plasmodium* species must be based on oxopurine nucleobases rather than nucleosides, and accommodate, sequentially: (1) effective transport by hFNT in the erythrocyte membrane; (2) effective uptake by *Pf* ENT1; (3) efficient conversion by *Pf* HGXPRT to the corresponding mononucleotide; and (4) the further conversion to ATP or GTP analog.

Medicinal chemistry approaches are essential to exploit both the parasite's purine transporters (i.e., *Pf* ENTs) and the unique features of *P. falciparum* purine metabolism. This strategy involves designing purine nucleobase analogs that are both efficiently transported and metabolized by the parasite. For example, modifications of the hENT1 inhibitor draflazine resulted in analogs with varying affinities for ENT1 and ENT2 across different mammalian species (Baldwin et al., 2007), demonstrating the feasibility of tailoring analogs for selective recognition by orthologous transporters, even of closely related species. Building on this principle, el Kouni et al. (1999) demonstrated that 6-(4-nitrobenzyl)-mercaptopurine riboside (NBMPR) could be used to treat *T. gondii* infections within human cells. Mammalian ENTs are historically distinguished based on their differential sensitivity to NBMPR: hENT1 (equilibrative/sensitive, *e.s*.), is sensitive to inhibition by sub-nanomolar concentrations of NBMPR, whereas hENT2 (equilibrative/insensitive, *e.i*.) requires micromolar to millimolar concentrations of NBMPR for complete inhibition (Baldwin et al., 2004; Griffiths et al., 1997). Notably, NBMPR is not ordinarily taken up by human cells, being a selective inhibitor rather than substrate of hENT1. el Kouni et al. (1999) concluded that *T. gondii* alters host cell membrane permeability, equivalent to the *Plasmodium* new permeation pathways, and so facilitates NBMPR uptake. This selective uptake of NBMPR was reinforced by the intracellular phosphorylation of the analog by *T. gondii* to its 5′-monophosphate, maintaining the concentration gradient for the free nucleoside. This example underscores the potential for purine analogs to be optimized for selective targeting of both the parasite transport and metabolic systems. Given that *P. falciparum* and *T. gondii* are both apicomplexan parasites, the safe, selective use of NBMPR in *T. gondii* further supports the plausibility of developing purine-based therapies that are selectively taken up by the *Pf* ENTs in malaria treatment, yet effective in penetrating the host cells—and potentially only by *Plasmodium*-infected cells, just like NBMPR was taken up selectively by *Toxoplasma*-infected host cells and nutrients are selectively accumulated in infected *Plasmodium*-erythrocytes by the new permeation pathways.

Although purine analogs have demonstrated potent antiparasitic effects (Al Safarjalani et al., 2008; Hulpia et al., 2019b; Mabille et al., 2022; Sosa et al., 2019; Tashie et al., 2025b; Ting et al., 2005), as a chemical class, their reputation for toxicity to human cells, particularly during cancer chemotherapy, remains a significant concern (Berman et al., 1985; Kim et al., 2019; Tseligka et al., 2023; Wang et al., 2016). However, the toxicity observed in cancer treatments often results from prolonged administration of these agents, and at high dosage. In contrast, antimalarial treatments typically involve short-term therapeutic regimens which are less likely to result in the severe side effects associated with long-term chemotherapy. More importantly, the cytotoxicity of anticancer nucleoside prodrugs is derived from their activation, i.e., phosphorylation in cancer cells, and eventual incorporation into nucleic acids, and avoiding the activation by human metabolic enzymes is certainly possible. A classic example is the acyclic class of antiviral guanosine analogs acyclovir, ganciclovir and valacyclovir that require activation by virus-encoded enzymes, thus limiting their toxicity to infected cells (Darby et al., 1981). This pharmacological distinction opens a path for designing purine analogs that are selectively activated within the parasite infected erythrocytes and hence minimizing host toxicity.

## 5 Conclusion

The absolute dependence of *P. falciparum* on the purine salvage pathway for proliferation and survival provides a compelling opportunity to target this pathway in the development of antimalarial drugs, particularly in the face of rising resistance to current therapies. The most effective approach will be to exploit *Pf* ENTs as conduits for purine-based analogs that act as “subversive” substrates of parasite purine-metabolizing enzymes, designed with greater sensitivity for parasite enzymes over human homologs. Purine analogs that efficiently enter *P. falciparum*-infected erythrocytes, reach *Pf* ENTs, and undergo selective activation within the parasite will disrupt purine metabolism and nucleic acid synthesis, ultimately leading to parasite death.

By integrating structural insights into *Pf* ENTs and key enzymes of *P. falciparum* purine metabolism with advanced medicinal chemistry techniques, it becomes feasible to develop purine-based therapies that exploit the distinct substrate affinities and structural properties of the parasite transporters. Such therapies could provide effective and safe antimalarial treatments, minimizing off-target effects in the host while addressing the urgent need for novel interventions that are not cross-resistant with current antimalarials. Structure-activity relationship studies and molecular docking simulations will be critical in guiding the target-based design of analogs optimized for *Pf* ENT1-mediated uptake and selective activation within *P. falciparum*.

In this context, recent work by Tashie et al. (2025b) identified several guanine derivative, 8-azaguanine, 7-deazaguanine, and 6-thioguanine, that inhibited the *in vitro* growth of *P. falciparum* at low micromolar concentrations. These compounds provide promising starting points for *Pf* ENT1-targeted drug development. Future medicinal chemistry approaches should focus on optimizing the antiplasmodial potency of these analogs while improving selectivity. A logical early exploration would involve combining the potent active modifications to guanine, for instance, 6-thio, 8-azaguanine, 6-thio, 7-deazaguanine, and 7-deaza, 8-azaguanine.

## References

[B1] Al Safarjalani O. N. Rais R. H. Kim Y. A. Chu C. K. Naguib F. N. el Kouni M. H. (2008). 7-Deaza-6-benzylthioinosine analogues as subversive substrate of *Toxoplasma gondii* adenosine kinase: activities and selective toxicities. Biochem. Pharmacol. 76, 958–966. doi: 10.1016/j.bcp.2008.07.035 18755159 PMC2581922

[B2] Aldfer M. M. Alfayez I. A. Elati H. A. A. Gayen N. Elmahallawy E. K. Milena Murillo A. . (2022a). The *Trypanosoma cruzi Tc*rNT2 nucleoside transporter is a conduit for the uptake of 5-F2′-deoxyuridine and tubercidin analogues. Molecules 27:8045. doi: 10.3390/molecules27228045 36432150 PMC9693223

[B3] Aldfer M. M. AlSiari T. A. Elati H. A. A. Natto M. J. Alfayez I. A. Campagnaro G. D. . (2022b). Nucleoside transport and nucleobase uptake null mutants in *Leishmania mexicana* for the routine expression and characterization of purine and pyrimidine transporters. Int. J. Mol. Sci. 23:8139. doi: 10.3390/ijms23158139 35897714 PMC9331716

[B4] Aldfer M. M. Hulpia F. van Calenbergh S. De Koning H. P. (2024). Mapping the transporter-substrate interactions of the *Trypanosoma cruz*i NB1 nucleobase transporter reveals the basis for its high affinity and selectivity for hypoxanthine and guanine and lack of nucleoside uptake. Mol. Biochem. Parasitol. 258:111616. doi: 10.1016/j.molbiopara.2024.111616 38401850

[B5] Ali J. A. Creek D. J. Burgess K. Allison H. C. Field M. C. Mäser P. . (2013). Pyrimidine salvage in *Trypanosoma brucei* bloodstream forms and the trypanocidal action of halogenated pyrimidines. Mol. Pharmacol. 83, 439–453. doi: 10.1124/mol.112.082321 23188714 PMC4857052

[B6] Al-Salabi M. I. de Koning H. P. (2005). Purine nucleobase transport in amastigotes of *Leishmania mexicana*: involvement in allopurinol uptake. Antimicrob. Agents Chemother. 49, 3682–3689. doi: 10.1128/AAC.49.9.3682-3689.2005 16127040 PMC1195421

[B7] Al-Salabi M. I. Wallace L. J. M. Lüscher A. Mäser P. Candlish D. Rodenko B. . (2007). Molecular interactions underlying the unusually high adenosine affinity of a novel *Trypanosoma brucei* nucleoside transporter. Mol. Pharmacol. 71, 921–929. doi: 10.1124/mol.106.031559 17185380

[B8] Alzahrani K. J. H. Ali J. A. M. Eze A. A. Looi W. L. Tagoe D. N. A. Creek D. J. . (2017). Functional and genetic evidence that nucleoside transport is highly conserved in *Leishmania* species: implications for pyrimidine-based chemotherapy. Int. J. Parasitol. Drugs Drug Resist. 7, 206–226. doi: 10.1016/j.ijpddr.2017.04.003 28453984 PMC5407577

[B9] Arora A. Deniskin R. Sosa Y. Nishtala S. N. Henrich P. P. Kumar T. R. . (2016). Substrate and inhibitor specificity of the *Plasmodium berghei* equilibrative nucleoside transporter type 1. Mol. Pharmacol. 89, 678–685. doi: 10.1124/mol.115.101386 27048953 PMC4885503

[B10] Assefa A. Fola A. A. Tasew G. (2024). Emergence of *Plasmodium falciparum* strains with artemisinin partial resistance in East Africa and the Horn of Africa: is there a need to panic? Malar. J. 23:34. doi: 10.1186/s12936-024-04848-8 38273360 PMC10809756

[B11] Baldwin S. A. Beal P. R. Yao S. Y. M. King A. E. Cass C. E. Young J. D. (2004). The equilibrative nucleoside transporter family, SLC29. Pflügers Arch. 447, 735–743. doi: 10.1007/s00424-003-1103-2 12838422

[B12] Baldwin S. A. McConkey G. A. Cass C. E. Young J. D. (2007). Nucleoside transport as a potential target for chemotherapy in malaria. Curr. Pharm. Des. 13, 569–580. doi: 10.2174/138161207780162845 17346175

[B13] Balikagala B. Fukuda N. Ikeda M. Katuro O. T. Tachibana S. I. Yamauchi M. . (2021). Evidence of artemisinin-resistant malaria in Africa. N. Engl. J. Med. 385, 1163–1171. doi: 10.1056/NEJMoa2101746 34551228

[B14] Basco L. K. (2023). Cultivation of asexual intraerythrocytic stages of *Plasmodium falciparum*. Pathogens 12:900. doi: 10.3390/pathogens12070900 37513747 PMC10384318

[B15] Berg M. Van der Veken P. Goeminne A. Haemers A. Augustyns K. (2010). Inhibitors of the purine salvage pathway: a valuable approach for antiprotozoal chemotherapy? Curr. Med. Chem. 17, 2456–2481. doi: 10.2174/092986710791556023 20491648

[B16] Berman J. J. Tong C. Williams G. M. (1985). Toxicity of 6-thioguanine and 8-azaguanine to non-dividing liver cell cultures. Cell. Biol. Toxicol. 1, 67–73. doi: 10.1007/BF00717792 3917127

[B17] Berman P. A. Human L. Freese J. A. (1991). Xanthine oxidase inhibits growth of *Plasmodium falciparum* in human erythrocytes in vitro. J. Clin. Invest. 88, 1848–1855. doi: 10.1172/JCI115506 1752946 PMC295752

[B18] Borbone N. Piccialli G. Roviello G. N. Oliviero G. (2021). Nucleoside analogs and nucleoside precursors as drugs in the fight against SARS-CoV-2 and other coronaviruses. Molecules 26:986. doi: 10.3390/molecules26040986 33668428 PMC7918729

[B19] Büngener W. Nielsen G. (1968). [Nucleic acid metabolism in experimental malaria. 2. Incorporation of adenosine and hypoxanthine into the nucleic acids of malaria parasites (*Plasmodium berghei* and *Plasmodium vinckei*)]. Z. Tropenmed. Parasitol. 19, 185–197. 4878204

[B20] Burchmore R. J. Wallace L. J. Candlish D. Al-Salabi M. I. Beal P. R. Barrett M. P. . (2003). Cloning, heterologous expression, and *in situ* characterization of the first high affinity nucleobase transporter from a protozoan. J. Biol. Chem. 278, 23502–23507. doi: 10.1074/jbc.M301252200 12707261

[B21] Campagnaro G. D. de Koning H. P. (2020). Purine and pyrimidine transporters of pathogenic protozoa—conduits for therapeutic agents. Med. Res. Rev. 40, 1679–1714. doi: 10.1002/med.21667 32144812

[B22] Carter N. S. Barrett M. P. de Koning H. P. (1999). A drug resistance determinant in *Trypanosoma brucei*. Trends Microbiol. 7, 469–471. doi: 10.1016/S0966-842X(99)01643-1 10603477

[B23] Carter N. S. Ben Mamoun C. Liu W. Silva E. O. Landfear S. M. Goldberg D. E. . (2000a). Isolation and functional characterization of the *Pf* NT1 nucleoside transporter gene from *Plasmodium falciparum*. J. Biol. Chem. 275, 10683–10691. doi: 10.1074/jbc.275.14.10683 10744765

[B24] Carter N. S. Drew M. E. Sanchez M. Vasudevan G. Landfear S. M. Ullman B. (2000b). Cloning of a novel inosine-guanosine transporter gene from *Leishmania donovani* by functional rescue of a transport-deficient mutant. J. Biol. Chem. 275, 20935–20941. doi: 10.1074/jbc.M002418200 10783393

[B25] Carter N. S. Rager N. Ullman B. (2003). “Purine and pyrimidine transport and metabolism,” in Molecular Medical Parasitology, eds. J. J. Marr, T. W. Nilsen and R. W. Komuniecki (London: Academic Press), 197–223. doi: 10.1016/B978-012473346-6/50012-0

[B26] Casali E. Berni P. Spisni A. Baricchi R. Pertinhez T.A. (2016). Hypoxanthine: a new paradigm to interpret the origin of transfusion toxicity. Blood Transfus 14, 555–556. doi: 10.2450/2015.0177-15 26674829 PMC5111384

[B27] Cassera B. M. Zhang Y. Hazleton Z. K. Schramm L. V. (2011a). Purine and pyrimidine pathways as targets in *Plasmodium falciparum*. Curr. Top. Med. Chem. 11, 2103–2115. doi: 10.2174/156802611796575948 21619511 PMC3319363

[B28] Cassera M. B. Hazleton K. Z. Merino E. F. Obaldia N. Ho M. C. Murkin A. S. . (2011b). *Plasmodium falciparum* parasites are killed by a transition state analogue of purine nucleoside phosphorylase in a primate animal model. PLoS One 6:e26916. doi: 10.1371/journal.pone.0026916 22096507 PMC3214022

[B29] Chen X. Tian C. He Y. Li Y. Zhou Y. Wang X. . (2025). Substrate and inhibitor specificity of *Plasmodium* nucleoside transporters ENT1 orthologs. J. Biol. Chem. 301:108115. doi: 10.1016/j.jbc.2024.108115 39725030 PMC11787452

[B30] Cheviet T. Lefebvre-Tournier I. Wein S. Peyrottes S. (2019). *Plasmodium* purine metabolism and its inhibition by nucleoside and nucleotide analogues. J. Med. Chem. 62, 8365–8391. doi: 10.1021/acs.jmedchem.9b00182 30964283

[B31] Conrad M. D. Asua V. Garg S. Giesbrecht D. Niaré K. Smith S. . (2023). Evolution of partial resistance to artemisinins in malaria parasites in Uganda. N. Engl. J. Med. 389, 722–732. doi: 10.1056/NEJMoa2211803 37611122 PMC10513755

[B32] Counihan N. A. Modak J. K. de Koning-Ward T. F. (2021). How malaria parasites acquire nutrients from their host. Front. Cell. Dev. Biol. 9:649184. doi: 10.3389/fcell.2021.649184 33842474 PMC8027349

[B33] Daily J. P. Parikh S. (2025). Malaria. N. Engl. J. Med. 392, 1320–1333. doi: 10.1056/NEJMra2405313 40174226 PMC12331251

[B34] Darby G. Field H. J. Salisbury S. A. (1981). Altered substrate specificity of herpes simplex virus thymidine kinase confers acyclovir-resistance. Nature 289, 81–83. doi: 10.1038/289081a0 6256650

[B35] de Koning H. P. Bridges D. J. Burchmore R. J. S. (2005). Purine and pyrimidine transport in pathogenic protozoa: from biology to therapy. FEMS Microbiol. Rev. 29, 987–1020. doi: 10.1016/j.femsre.2005.03.004 16040150

[B36] de Koning H. P. Jarvis S. M. (1997). Hypoxanthine uptake through a purine-selective nucleobase transporter in *Trypanosoma brucei brucei* procyclic cells is driven by protonmotive force. Eur. J. Biochem. 247, 1102–1110. doi: 10.1111/j.1432-1033.1997.01102.x 9288936

[B37] de Koning H. P. Jarvis S. M. (1998). A highly selective, high-affinity transporter for uracil in *Trypanosoma brucei bruce*i: evidence for proton-dependent transport. Biochem. Cell. Biol. 76, 853–858. doi: 10.1139/o98-086 10353720

[B38] de Koning H. P. Jarvis S. M. (1999). Adenosine transporters in bloodstream forms of *Trypanosoma brucei bruce*i: substrate recognition motifs and affinity for trypanocidal drugs. Mol. Pharmacol. 56, 1162–1170. doi: 10.1016/S0026-895X(24)12381-4 10570043

[B39] de Koning H. P. Macleod A. Barrett M. P. Cover B. Jarvis S. M. (2000a). Further evidence for a link between melarsoprol resistance and P2 transporter function in African trypanosomes. Mol. Biochem. Parasitol. 106, 181–185. doi: 10.1016/S0166-6851(99)00206-6 10743623

[B40] de Koning H. P. Watson C. J. Jarvis S. M. (1998). Characterization of a nucleoside/proton symporter in procyclic *Trypanosoma brucei brucei*. J. Biol. Chem. 273, 9486–9494. doi: 10.1074/jbc.273.16.9486 9545276

[B41] de Koning H. P. Watson C. J. Sutcliffe L. Jarvis S. M. (2000b). Differential regulation of nucleoside and nucleobase transporters in *Crithidia fasciculata* and *Trypanosoma brucei brucei*. Mol. Biochem. Parasitol. 106, 93–107. doi: 10.1016/S0166-6851(99)00203-0 10743614

[B42] Delespaux V. de Koning H. P. (2013). “Transporters in anti-parasitic drug development and resistance,” in Trypanosomatid Diseases: Molecular Routes to Drug Discovery, eds. T. Jäger, O. Koch and L. Flohé (Weinheim Germany: Wiley-VCH Verlag GmbH and Co. KGaA), 335–349. doi: 10.1002/9783527670383.ch18

[B43] Deniskin R. Frame I. J. Sosa Y. Akabas M. H. (2016). Targeting the *Plasmodium vivax* equilibrative nucleoside transporter 1 (*Pv*ENT1) for antimalarial drug development. Int. J. Parasitol. Drugs Drug Resist. 6, 1–11. doi: 10.1016/j.ijpddr.2015.11.003 26862473 PMC4706624

[B44] Domin B. A. Mahony W. B. Zimmerman T. P. (1988). Purine nucleobase transport in human erythrocytes. Reinvestigation with a novel “inhibitor-stop” assay. J. Biol. Chem. 263, 9276–9284. doi: 10.1016/S0021-9258(19)76536-3 3379069

[B45] Dondorp A. M. Nosten F. Yi P. Das D. Phyo A. P. Tarning J. . (2009). Artemisinin resistance in *Plasmodium falciparum* malaria. N. Engl. J. Med. 361, 455–467. doi: 10.1056/NEJMoa0808859 19641202 PMC3495232

[B46] Downie M. J. Saliba K. J. Bröer S. Howitt S. M. Kirk K. (2008). Purine nucleobase transport in the intraerythrocytic malaria parasite. Int. J. Parasitol. 38, 203–209. doi: 10.1016/j.ijpara.2007.07.005 17765902

[B47] Downie M. J. Saliba K. J. Howitt S. M. Bröer S. Kirk K. (2006). Transport of nucleosides across the *Plasmodium falciparum* parasite plasma membrane has characteristics of *Pf* ENT1. Mol. Microbiol. 60, 738–748. doi: 10.1111/j.1365-2958.2006.05125.x 16629674

[B48] Ducati R. G. Namanja-Magliano H. A. Schramm V. L. (2013). Transition-state inhibitors of purine salvage and other prospective enzyme targets in malaria. Future Med. Chem. 5, 1341–1360. doi: 10.4155/fmc.13.51 23859211 PMC3819805

[B49] Dudzinska W. Hlynczak A. J. Skotnicka E. Suska M. (2006). The purine metabolism of human erythrocytes. Biochemistry (Mosc.) 71, 467–475. doi: 10.1134/S0006297906050014 16732723

[B50] El Bissati K. Downie M. J. Kim S. K. Horowitz M. Carter N. Ullman B. . (2008). Genetic evidence for the essential role of *Pf* NT1 in the transport and utilization of xanthine, guanine, guanosine and adenine by *Plasmodium falciparum*. Mol. Biochem. Parasitol. 161, 130–139. doi: 10.1016/j.molbiopara.2008.06.012 18639591 PMC2612043

[B51] El Bissati K. Zufferey R. Witola W. H. Carter N. S. Ullman B. Ben Mamoun C. (2006). The plasma membrane permease *Pf* NT1 is essential for purine salvage in the human malaria parasite *Plasmodium falciparum*. Proc. Natl. Acad. Sci. 103, 9286–9291. doi: 10.1073/pnas.0602590103 16751273 PMC1482602

[B52] el Kouni M. H. (2003). Potential chemotherapeutic targets in the purine metabolism of parasites. Pharmacol. Ther. 99, 283–309. doi: 10.1016/S0163-7258(03)00071-8 12951162

[B53] el Kouni M. H. (2017). Pyrimidine metabolism in schistosomes: a comparison with other parasites and the search for potential chemotherapeutic targets. Comp. Biochem. Physiol. B Biochem. Mol. Biol. 213, 55–80. doi: 10.1016/j.cbpb.2017.07.001 28735972 PMC5593796

[B54] el Kouni M. H. Guarcello V. Al Safarjalani O. N. Naguib F. N. (1999). Metabolism and selective toxicity of 6-nitrobenzylthioinosine in *Toxoplasma gondii*. Antimicrob. Agents Chemother. 43, 2437–2443. doi: 10.1128/AAC.43.10.2437 10508021 PMC89497

[B55] Elati H. A. A. Goerner A. L. Martorelli Di Genova B. Sheiner L. de Koning H. P. (2023). Pyrimidine salvage in *Toxoplasma gondii* as a target for new treatment. Front. Cell. Infect. Microbiol. 13:1320160. doi: 10.3389/fcimb.2023.1320160 38162577 PMC10755004

[B56] Feng J. Y. (2018). Addressing the selectivity and toxicity of antiviral nucleosides. Antivir. Chem. Chemother. 26:2040206618758524. doi: 10.1177/2040206618758524 29534607 PMC5890540

[B57] Fiuza L. F. A. Batista D. G. J. Girão R. D. Hulpia F. Finamore-Araújo P. Aldfer M. M. . (2022). Phenotypic evaluation of nucleoside analogues against *Trypanosoma cruzi* infection: in vitro and in vivo approaches. Molecules 27:8087. doi: 10.3390/molecules27228087 36432189 PMC9695592

[B58] Fox B. A. Bzik D. J. (2020). “Biochemistry and metabolism of *Toxoplasma gondii*: purine and pyrimidine acquisition in *Toxoplasma gondii* and other *Apicomplexa*,” in Toxoplasma gondii, 3rd Edn, eds L. M. Weiss and K. Kim, (Cambridge, MA: Academic Press), 397–449. doi: 10.1016/B978-0-12-815041-2.00009-8

[B59] Frame I. J. Deniskin R. Arora A. Akabas M. H. (2015a). Purine import into malaria parasites as a target for antimalarial drug development. Ann. N. Y. Acad. Sci. 1342, 19–28. doi: 10.1111/nyas.12568 25424653 PMC4405406

[B60] Frame I. J. Deniskin R. Rinderspacher A. Katz F. Deng S. X. Moir R. D. . (2015b). Yeast-based high-throughput screen identifies *Plasmodium falciparum* equilibrative nucleoside transporter 1 inhibitors that kill malaria parasites. ACS Chem. Biol. 10, 775–783. doi: 10.1021/cb500981y 25602169 PMC4369170

[B61] Frame I. J. Merino E. F. Schramm V. L. Cassera M. B. Akabas M. H. (2012). Malaria parasite type 4 equilibrative nucleoside transporters (ENT4) are purine transporters with distinct substrate specificity. Biochem. J. 446, 179–190. doi: 10.1042/BJ20112220 22670848 PMC3756485

[B62] Galmarini C. M. Mackey J. R. Dumontet C. (2002). Nucleoside analogues and nucleobases in cancer treatment. Lancet Oncol. 3, 415–424. doi: 10.1016/S1470-2045(02)00788-X 12142171

[B63] Gardner M. J. Hall N. Fung E. White O. Berriman M. Hyman R. W. . (2002). Genome sequence of the human malaria parasite *Plasmodium falciparum*. Nature 419, 498–511. doi: 10.1038/nature01097 12368864 PMC3836256

[B64] Geiser F. Lüscher A. de Koning H. P. Seebeck T. Mäser P. (2005). Molecular pharmacology of adenosine transport in *Trypanosoma brucei*: P1/P2 revisited. Mol. Pharmacol. 68, 589–595. doi: 10.1124/mol.104.010298 15933219

[B65] Geraghty R. J. Aliota M. T. Bonnac L. F. (2021). Broad-spectrum antiviral strategies and nucleoside analogues. Viruses 13:667. doi: 10.3390/v13040667 33924302 PMC8069527

[B66] Gero A. M. O'Sullivan W. J. (1990). Purines and pyrimidines in malarial parasites. Blood Cells Mol. Dis. 16, 467–498. 2257323

[B67] Ghérardi A. Sarciron M. (2007). Molecules targeting the purine salvage pathway in Apicomplexan parasites. Trends Parasitol. 23, 384–389. doi: 10.1016/j.pt.2007.06.003 17574921

[B68] Ginsburg H. (2016). “The biochemistry of Plasmodium falciparum: an updated overview,” in Advances in Malaria Research, eds D. Gaur, C. E. Chitrnis and V. S. Chauhan (Hoboken, NJ: John Wiley and Sons, Inc), 219–290. doi: 10.1002/9781118493816.ch9

[B69] Ginsburg H. Kutner S. Krugliak M. Ioav-Cabantchik Z. (1985). Characterization of permeation pathways appearing in the host membrane of *Plasmodium falciparum* infected red blood cells. Mol. Biochem. Parasitol. 14, 313–322. doi: 10.1016/0166-6851(85)90059-3 3887158

[B70] Graf F. E. Ludin P. Wenzler T. Kaiser M. Brun R. Pyana P. P. . (2013). Aquaporin 2 mutations in *Trypanosoma brucei* gambiense field isolates correlate with decreased susceptibility to pentamidine and melarsoprol. PLoS Negl. Trop. Dis. 7:e2475. doi: 10.1371/journal.pntd.0002475 24130910 PMC3794916

[B71] Griffiths M. Beaumont N. Yao S. Y. M. Sundaram M. Boumah C. E. Davies A. . (1997). Cloning of a human nucleoside transporter implicated in the cellular uptake of adenosine and chemotherapeutic drugs. Nat. Med. 3, 89–93. doi: 10.1038/nm0197-89 8986748

[B72] Gutteridge W. E. Trigg P. I. (1970). Incorporation of radioactive prec/ursors into DNA and RNA of *Plasmodium knowlesi* in vitro. J. Protozool. 17, 89–96. doi: 10.1111/j.1550-7408.1970.tb05163.x 5420334

[B73] Hall S. T. Hillier C. J. Gero A. M. (1996). *Crithidia luciliae*: regulation of purine nucleoside transport by extracellular purine concentrations. Exp. Parasitol. 83, 314–321. doi: 10.1006/expr.1996.0079 8823248

[B74] Hruba L. Das V. Hajduch M. Dzubak P. (2023). Nucleoside-based anticancer drugs: mechanism of action and drug resistance. Biochem. Pharmacol. 215:115741. doi: 10.1016/j.bcp.2023.115741 37567317

[B75] Huang S. Bian Y. Huang C. Miao L. (2022). Is monitoring of the intracellular active metabolite levels of nucleobase and nucleoside analogs ready for precision medicine applications? Eur. J. Drug Metab. Pharmacokinet. 47, 761–775. doi: 10.1007/s13318-022-00786-5 35915365

[B76] Hulpia F. Campagnaro G. D. Scortichini M. Van Hecke K. Maes L. de Koning H. P. . (2019a). Revisiting tubercidin against kinetoplastid parasites: aromatic substitutions at position 7 improve activity and reduce toxicity. Eur. J. Med. Chem. 164, 689–705. doi: 10.1016/j.ejmech.2018.12.050 30677668

[B77] Hulpia F. Mabille D. Campagnaro G. D. Schumann G. Maes L. Roditi I. . (2019b). Combining tubercidin and cordycepin scaffolds results in highly active candidates to treat late-stage sleeping sickness. Nat. Commun. 10:5564. doi: 10.1038/s41467-019-13522-6 31804484 PMC6895180

[B78] Jordheim L. P. Durantel D. Zoulim F. Dumontet C. (2013). Advances in the development of nucleoside and nucleotide analogues for cancer and viral diseases. Nat. Rev. Drug Discov. 12, 447–464. doi: 10.1038/nrd4010 23722347

[B79] Josling G. A. Llinás M. (2015). Sexual development in *Plasmodium* parasites: knowing when it's time to commit. Nat. Rev. Microbiol. 13, 573–587. doi: 10.1038/nrmicro3519 26272409

[B80] Juliano J. J. Giesbrecht D. J. Simkin A. Fola A. A. Lyimo B. M. Pereus D. . (2024). Prevalence of mutations associated with artemisinin partial resistance and sulfadoxine-pyrimethamine resistance in 13 regions in Tanzania in 2021: a cross-sectional survey. Lancet Microbe 5:100920. doi: 10.1016/S2666-5247(24)00160-5 39159629 PMC11464622

[B81] Kamzeeva P. N. Aralov A. V. Alferova V. A. Korshun V. A. (2023). Recent advances in molecular mechanisms of nucleoside antivirals. Curr. Issues Mol. Biol. 45, 6851–6879. doi: 10.3390/cimb45080433 37623252 PMC10453654

[B82] Kavishe R. A. Koenderink J. B. Alifrangis M. (2017). Oxidative stress in malaria and artemisinin combination therapy: pros and cons. FEBS J. 284, 2579–2591. doi: 10.1111/febs.14097 28467668

[B83] Keough D. T. Ng A.-L. Winzor D. T. Emmerson B. T. de Jersey J. (1999). Purification and characterization of *Plasmodium falciparum* hypoxanthine–guanine–xanthine phosphoribosyltransferase and comparison with the human enzyme. Mol. Biochem. Parasitol. 98, 29–41. doi: 10.1016/S0166-6851(98)00139-X 10029307

[B84] Kim N. Choi J. Song A. Choi W. Park H. Park S. . (2019). Direct potentiation of NK cell cytotoxicity by 8-azaguanine with potential antineoplastic activity. Int. Immunopharmacol. 67, 152–159. doi: 10.1016/j.intimp.2018.12.020 30551032

[B85] Kim Y. A. Sharon A. Chu C. K. Rais R. H. Al Safarjalani O. N. Naguib F. N. . (2008). Structure-activity relationships of 7-deaza-6-benzylthioinosine analogues as ligands of *Toxoplasma gondii* adenosine kinase. J. Med. Chem. 51, 3934–3945. doi: 10.1021/jm800201s 18563892

[B86] Kimenyi K. M. Akinyi M. Y. Mwikali K. Gilmore T. Mwangi S. Omer E. . (2024). Distinct transcriptomic signatures define febrile malaria depending on initial infective states, asymptomatic or uninfected. BMC Infect. Dis. 24:140. doi: 10.1186/s12879-024-08973-2 38287287 PMC10823747

[B87] King K. M. Damaraju V. L. Vickers M. F. Yao S. Y. Lang T. Tackaberry T. E. . (2006). A comparison of the transportability, and its role in cytotoxicity, of clofarabine, cladribine, and fludarabine by recombinant human nucleoside transporters produced in three model expression systems. Mol. Pharmacol. 69, 346–353. doi: 10.1124/mol.105.015768 16234483

[B88] Krungkrai S. R. Krungkrai J. (2016). Insights into the pyrimidine biosynthetic pathway of human malaria parasite *Plasmodium falciparum* as chemotherapeutic target. Asian Pac. J. Trop. Med. 9, 525–534. doi: 10.1016/j.apjtm.2016.04.012 27262062

[B89] Landfear S. M. Ullman B. Carter N. S. Sanchez M. A. (2004). Nucleoside and nucleobase transporters in parasitic protozoa. Eukaryot. Cell 3, 245–254. doi: 10.1128/EC.3.2.245-254.2004 15075255 PMC387651

[B90] Lin C. Gama D. Batista J. Mazzeti A. L. Girão R. D. de Oliveira G. M. . (2022). N6-modification of 7-deazapurine nucleoside analogues as anti-*Trypanosoma cruzi* and anti-*Leishmania* agents: structure-activity relationship exploration and *in vivo* evaluation. Eur. J. Med. Chem. 231:114165. doi: 10.1016/j.ejmech.2022.114165 35144125

[B91] Lingelbach K. Kirk K. Rogerson S. Langhorne J. Carucci D. J. Waters A. (2004). Molecular approaches to malaria. Mol. Microbiol. 54, 575–587. doi: 10.1111/j.1365-2958.2004.04362.x 15491351

[B92] Lüscher A. de Koning H. P. Mäser P. (2007). Chemotherapeutic strategies against *Trypanosoma brucei*: drug targets vs. drug targeting. Curr. Pharm. Des. 13, 555–567. doi: 10.2174/138161207780162809 17346174

[B93] Mabille D. Ilbeigi K. Hendrickx S. Ungogo M. A. Hulpia F. Lin C. . (2022). Nucleoside analogues for the treatment of animal trypanosomiasis. Int. J. Parasitol. Drugs Drug Resist. 19, 21–30. doi: 10.1016/j.ijpddr.2022.05.001 35567803 PMC9111543

[B94] Martin J. L. Yates P. A. Soysa R. Alfaro J. F. Yang F. Burnum-Johnson K. E. . (2014). Metabolic reprogramming during purine stress in the protozoan pathogen *Leishmania donovani*. PLoS Pathog. 10:e1003938. doi: 10.1371/journal.ppat.1003938 24586154 PMC3937319

[B95] Mäser P. Sütterlin C. Kralli A. Kaminsky R. (1999). A nucleoside transporter from *Trypanosoma brucei* involved in drug resistance. Science 285, 242–244. doi: 10.1126/science.285.5425.242 10398598

[B96] Matovu E. Stewart M. L. Geiser F. Brun R. Mäser P. Wallace L. J. . (2003). Mechanisms of arsenical and diamidine uptake and resistance in *Trypanosoma brucei*. Eukaryot. Cell 2, 1003–1008. doi: 10.1128/EC.2.5.1003-1008.2003 14555482 PMC219364

[B97] Menezes S. A. Cardoso F. G. Venturi C. R. Barden A. T. Tasca T. (2025). Adenosine deprivation modulates purine metabolism and enhances *Trichomonas vaginalis* cytotoxicity. Biochimie 239, 163–176. doi: 10.1016/j.biochi.2025.08.015 40850382

[B98] Mihreteab S. Anderson K. Fuente I. M. Sutherland C. J. Smith D. Cunningham J. . (2025). The spread of molecular markers of artemisinin partial resistance and diagnostic evasion in Eritrea: a retrospective molecular epidemiology study. Lancet Microbe 6:100930. doi: 10.1016/S2666-5247(24)00172-1 39653047 PMC11798904

[B99] Muggia F. Diaz I. Peters G. J. (2012). Nucleoside and nucleobase analogs in cancer treatment: not only sapacitabine, but also gemcitabine. Expert Opin. Investig. Drugs 21, 403–408. doi: 10.1517/13543784.2012.666236 22404148

[B100] Munday J. C. Tagoe D. N. Eze A. A. Krezdorn J. A. Rojas López K. E. Alkhaldi A. A. . (2015). Functional analysis of drug resistance-associated mutations in the *Trypanosoma brucei* adenosine transporter 1 (*Tb*AT1) and the proposal of a structural model for the protein. Mol. Microbiol. 96, 887–900. doi: 10.1111/mmi.12979 25708978 PMC4755147

[B101] Natto M. J. Hulpia F. Kalkman E. R. Baillie S. Alhejeli A. Miyamoto Y. . (2021a). Deazapurine Nucleoside Analogues for the Treatment of *Trichomonas vaginalis*. ACS Infect. Dis. 7, 1752–1764. doi: 10.1021/acsinfecdis.1c00075 33974405 PMC9586919

[B102] Natto M. J. Miyamoto Y. Munday J. C. AlSiari T. A. Al-Salabi M. I. Quashie N. B. . (2021b). Comprehensive characterization of purine and pyrimidine transport activities in *Trichomonas vaginalis* and functional cloning of a trichomonad nucleoside transporter. Mol. Microbiol. 116, 1489–1511. doi: 10.1111/mmi.14840 34738285 PMC8688338

[B103] Noedl H. Se Y. Schaecher K. Smith B. L. Socheat D. Fukuda M. M. (2008). Evidence of artemisinin-resistant malaria in western Cambodia. N. Engl. J. Med. 359, 2619–2620. doi: 10.1056/NEJMc0805011 19064625

[B104] Nosten F. White N. J. (2007). Artemisinin-based combination treatment of falciparum malaria. Am. J. Trop. Med. Hyg. 77(6 Suppl), 181–192. doi: 10.4269/ajtmh.2007.77.181 18165491

[B105] Ogwang R. Osoti V. Wamae K. Ndwiga L. Muteru K. Ningwa A. . (2024). A retrospective analysis of *P. falciparum* drug resistance markers detects an early (2016/17) high prevalence of the K13 C469Y mutation in asymptomatic infections in Northern Uganda. Antimicrob. Agents Chemother. 68:e0157623. doi: 10.1128/aac.01576-23 39136465 PMC11382623

[B106] Ortiz D. Sanchez M. A. Koch H. P. Larsson H. P. Landfear S. M. (2009). An acid-activated nucleobase transporter from *Leishmania major*. J. Biol. Chem. 284, 16164–16169. doi: 10.1074/jbc.M109.006718 19366701 PMC2713545

[B107] Parker M. D. Hyde R. J. Yao S. Y. M. Mcrobert L. Cass C. E. Young J. D. . (2000). Identification of a nucleoside/nucleobase transporter from *Plasmodium falciparum*, a novel target for anti-malarial chemotherapy. Biochem. J. 349, 67–75. doi: 10.1042/bj3490067 10861212 PMC1221121

[B108] Pastor-Anglada M. Pérez-Torras S. (2015). Nucleoside transporter proteins as biomarkers of drug responsiveness and drug targets. Front. Pharmacol. 6:13. doi: 10.3389/fphar.2015.00013 25713533 PMC4322540

[B109] Pastuch-Gawołek G. Gillner D. Król E. Walczak K. Wandzik I. (2019). Selected nucleos(t)ide-based prescribed drugs and their multi-target activity. Eur. J. Pharmacol. 865:172747. doi: 10.1016/j.ejphar.2019.172747 31634460 PMC7173238

[B110] Phillips M. A. Burrows J. N. Manyando C. van Huijsduijnen R. H. Van Voorhis W. C. Wells T. N. C. (2017). Malaria. Nat. Rev. Dis. Primers 3:17050. doi: 10.1038/nrdp.2017.50 28770814

[B111] Pruijssers A. J. Denison M. R. (2019). Nucleoside analogues for the treatment of coronavirus infections. Curr. Opin. Virol. 35, 57–62. doi: 10.1016/j.coviro.2019.04.002 31125806 PMC7102703

[B112] Quashie N. Dorin-Semblat D. Bray P. Biagini G. Doerig C. Ranford-Cartwright L. . (2008). A comprehensive model of purine uptake by the malaria parasite *Plasmodium falciparum*: identification of four purine transport activities in intraerythrocytic parasites. Biochem. J. 411, 287–295. doi: 10.1042/BJ20071460 18215139

[B113] Quashie N. B. Ranford-Cartwright L. C. de Koning H. P. (2010). Uptake of purines in *Plasmodium falciparum*-infected human erythrocytes is mostly mediated by the human equilibrative nucleoside transporter and the human facilitative nucleobase transporter. Malar. J. 9, 36. doi: 10.1186/1475-2875-9-36 20113503 PMC2825241

[B114] Queen S. A. Vander-Jagt D. Reyes P. (1988). Properties and substrate specificity of a purine phosphoribosyltransferase from the human malaria parasite, *Plasmodium falciparum*. Mol. Biochem. Parasitol. 30, 123–134. doi: 10.1016/0166-6851(88)90105-3 3050515

[B115] Rehan S. Shahid S. Salminen T. A. Jaakola V. Paavilainen V. O. (2019). Current progress on equilibrative nucleoside transporter function and inhibitor design. SLAS Discov. 24, 953–968. doi: 10.1177/2472555219870123 31503511

[B116] Riegelhaupt P. M. Frame I. J. Akabas M. H. (2010). Transmembrane segment 11 appears to line the purine permeation pathway of the *Plasmodium falciparum* equilibrative nucleoside transporter 1 (*Pf* ENT1). J. Biol. Chem. 285, 17001–17010. doi: 10.1074/jbc.M110.115758 20335165 PMC2878030

[B117] Rosenthal P. J. Asua V. Bailey J. A. Conrad M. D. Ishengoma D. S. Kamya M. R. . (2024). The emergence of artemisinin partial resistance in Africa: how do we respond? Lancet Infect. Dis. 24, e591–e600. doi: 10.1016/S1473-3099(24)00141-5 38552654 PMC12954456

[B118] Rottenberg M. E. Masocha W. Ferella M. Petitto-Assis F. Goto H. Kristensson K. . (2005). Treatment of African trypanosomiasis with cordycepin and adenosine deaminase inhibitors in a mouse model. J. Infect. Dis. 192, 1658–1665. doi: 10.1086/496896 16206083

[B119] Sibomana O. Bukuru J. Saka S. A. Uwizeyimana M. G. Kihunyu A. M. Obianke A. . (2025). Routine malaria vaccination in Africa: a step toward malaria eradication? Malar. J. 24:1. doi: 10.1186/s12936-024-05235-z 39757179 PMC11702236

[B120] Sosa Y. Deniskin R. Frame I. J. Steiginga M. S. Bandyopadhyay D. Graybill T. L. . (2019). Identification via a parallel hit progression strategy of improved small molecule inhibitors of the malaria purine uptake transporter that inhibit *Plasmodium falciparum* parasite proliferation. ACS Infect. Dis. 5, 1738–1753. doi: 10.1021/acsinfecdis.9b00168 31373203 PMC7171677

[B121] Stewart M. L. Burchmore R. J. Clucas C. Hertz-Fowler C. Brooks K. Tait A. . (2010). Multiple genetic mechanisms lead to loss of functional *Tb*AT1 expression in drug-resistant trypanosomes. Eukaryot. Cell 9, 336–343. doi: 10.1128/EC.00200-09 19966032 PMC2823006

[B122] Straimer J. Gandhi P. Renner K. C. Schmitt E. K. (2022). High prevalence of *Plasmodium falciparum* K13 mutations in Rwanda is associated with slow parasite clearance after treatment with artemether-lumefantrine. J. Infect. Dis. 225, 1411–1414. doi: 10.1093/infdis/jiab352 34216470 PMC9016418

[B123] Tashie W. de Koning H. P. Duah-Quashie N. O. Quashie N. B. (2025a). Genetic diversity of *Plasmodium falciparum* equilibrative nucleoside transporters *Pf* ENT1 and *Pf* ENT4: implications for purine-based antimalarial drug development. Int. J. Parasitol. 2025:104760. doi: 10.1016/j.ijpara.2025.12.005 41419157

[B124] Tashie W. de Koning H. P. Duah-Quashie N. O. Quashie N. B. (2025b). Guanine derivatives as promising candidates for the development of purine-based antimalarial drugs. Front. Parasitol. 4:1634209. doi: 10.3389/fpara.2025.1634209 40810137 PMC12343510

[B125] Tebben K. Yirampo S. Coulibaly D. Koné A. K. Laurens M. B. Stucke E. M. . (2024). Gene expression analyses reveal differences in children's response to malaria according to their age. Nat. Commun. 15:2021. doi: 10.1038/s41467-024-46416-3 38448421 PMC10918175

[B126] Tewari S. G. Rajaram K. Schyman P. Swift R. Reifman J. Prigge S. T. . (2019). Short-term metabolic adjustments in *Plasmodium falciparum* counter hypoxanthine deprivation at the expense of long-term viability. Malar J. 18:86. doi: 10.1186/s12936-019-2720-3 30890151 PMC6423861

[B127] Theodoridis L. Carvalho T. G. (2025). Antimalarial drug resistance and drug discovery: learning from the past to innovate the future. Int. J. Parasitol. Drugs Drug Resist. 28:100602. doi: 10.1016/j.ijpddr.2025.100602 40680501 PMC12296534

[B128] Thomas S. L. Egée S. (2005). “New permeation pathways,” in Molecular Approaches to Malaria, ed I. W. Sherman. (Washington, D.C.: ASM Press), 384–396. doi: 10.1128/9781555817558.ch20

[B129] Thomson J. M. Lamont I. L. (2019). Nucleoside analogues as antibacterial agents. Front. Microbiol. 10:952. doi: 10.3389/fmicb.2019.00952 31191461 PMC6540614

[B130] Ting L. Shi W. Lewandowicz A. Singh V. Mwakingwe A. Birck M. R. . (2005). Targeting a novel *Plasmodium falciparum* purine recycling pathway with specific immucillins. J. Biol. Chem. 280, 9547–9554. doi: 10.1074/jbc.M412693200 15576366

[B131] Tseligka E. D. Conzelmann S. Cambet Y. Schaer T. Negro F. Clément S. (2023). Identification of selective hepatitis delta virus ribozyme inhibitors by high-throughput screening of small molecule libraries. JHEP Reports 5:100652. doi: 10.1016/j.jhepr.2022.100652 36704052 PMC9871325

[B132] Ungogo M. A. Aldfer M. M. Natto M. J. Zhuang H. Chisholm R. Walsh K. . (2023). Cloning and characterization of *Trypanosoma congolense* and *T. vivax* nucleoside transporters reveal the potential of P1-type carriers for the discovery of broad-spectrum nucleoside-based therapeutics against animal African trypanosomiasis. Int. J. Mol. Sci. 24:3144. doi: 10.3390/ijms24043144 36834557 PMC9960827

[B133] Uwimana A. Umulisa N. Venkatesan M. Svigel S. S. Zhou Z. Munyaneza T. . (2021). Association of *Plasmodium falciparum* kelch13 R561H genotypes with delayed parasite clearance in Rwanda: an open-label, single-arm, multicentre, therapeutic efficacy study. Lancet Infect. Dis. 21, 1120–1128. doi: 10.1016/S1473-3099(21)00142-0 33864801 PMC10202849

[B134] Vaskó B. Juhász V. Tóth B. Kurunczi A. Fekete Z. Krisjanis Zolnerciks J. . (2019). Inhibitor selectivity of CNTs and ENTs. Xenobiotica 49, 840–851. doi: 10.1080/00498254.2018.1501832 30022699

[B135] Vasudevan G. Carter N. S. Drew M. E. Beverley S. M. Sanchez M. A. Seyfang A. . (1998). Cloning of *Leishmania* nucleoside transporter genes by rescue of a transport-deficient mutant. Proc. Natl. Acad. Sci. 95, 9873–9878. doi: 10.1073/pnas.95.17.9873 9707568 PMC21429

[B136] Vickers M. F. Zhang J. Visser F. Tackaberry T. Robins M. J. Nielsen L. P. C. . (2004). Uridine recognition motifs of human equilibrative nucleoside transporters 1 and 2 produced in *Saccharomyces cerevisiae*. Nucleosides Nucleotides Nucleic Acids 23, 361–373. doi: 10.1081/NCN-120028333 15043160

[B137] Vodnala M. Fijolek A. Rofougaran R. Mosimann M. Mäser P. Hofer A. (2008). Adenosine kinase mediates high affinity adenosine salvage in *Trypanosoma brucei*. J. Biol. Chem. 283, 5380–5388. doi: 10.1074/jbc.M705603200 18167353

[B138] Vodnala S. K. Lundbäck T. Yeheskieli E. Sjöberg B. Gustavsson A. L. Svensson R. . (2013). Structure-activity relationships of synthetic cordycepin analogues as experimental therapeutics for African trypanosomiasis. J. Med. Chem. 56, 9861–9873. doi: 10.1021/jm401530a 24283924

[B139] Wallace L. J. M. Candlish D. De Koning H. (2002). Different substrate recognition motifs of human and *Trypanosome* nucleobase transporters: selective uptake of purine antimetabolites. J. Biol. Chem. 277, 26149–26156. doi: 10.1074/jbc.M202835200 12004061

[B140] Wang C. Yu L. Zhang J. Zhou Y. Sun B. Xiao Q. . (2023). Structural basis of the substrate recognition and inhibition mechanism of *Plasmodium falciparum* nucleoside transporter *Pf* ENT1. Nat. Commun. 14:1727. doi: 10.1038/s41467-023-37411-1 36977719 PMC10050424

[B141] Wang S. Liu J. C. Kim D. Datti A. Zacksenhaus E. (2016). Targeted pten deletion plus p53-R270H mutation in mouse mammary epithelium induces aggressive claudin-low and basal-like breast cancer. Breast Cancer Res. 18:9. doi: 10.1186/s13058-015-0668-y 26781438 PMC4717616

[B142] Ward K. E. Fidock D. A. Bridgford J. L. (2022). *Plasmodium falciparum* resistance to artemisinin-based combination therapies. Curr. Opin. Microbiol. 69:102193. doi: 10.1016/j.mib.2022.102193 36007459 PMC9847095

[B143] Webster H. K. Whaun J. M. Walker M. D. Bean T. L. (1984). Synthesis of adenosine nucleotides from hypoxanthine by human malaria parasites (*Plasmodium falciparum*) in continuous erythrocyte culture: inhibition by hadacidin but not alanosine. Biochem. Pharmacol. 33, 1555–1557. doi: 10.1016/0006-2952(84)90427-1 6375681

[B144] WHO (2025a). Malaria: Artemisinin Partial Resistance. Available online at: https://www.who.int/news-room/questions-and-answers/item/artemisinin-resistance (Accessed January 22, 2026).

[B145] WHO (2025b). WHO Guidelines for Malaria. Available online at: https://www.who.int/publications/i/item/guidelines-for-malaria (Accessed December 15, 2025).

[B146] WHO (2025c). World Malaria Report 2025: Addressing the Threat of Antimalarial Drug Resistance. Available online at: https://www.who.int/teams/global-malaria-programme/reports/world-malaria-report-2025 (Accessed December 12, 2025).

[B147] Wichers J. S. Tonkin-Hill G. Thye T. Krumkamp R. Kreuels B. Strauss J. . (2021). Common virulence gene expression in adult first-time infected malaria patients and severe cases. eLife 10:e69040. doi: 10.7554/eLife.69040.sa2 33908865 PMC8102065

[B148] Zhang M. Wang C. Otto T. D. Oberstaller J. Liao X. Adapa S. R. . (2018). Uncovering the essential genes of the human malaria parasite *Plasmodium falciparum* by saturation mutagenesis. Science 360:eaap7847. doi: 10.1126/science.aap7847 29724925 PMC6360947

